# On the Exploration of Quantum Polar Stabilizer Codes and Quantum Stabilizer Codes with High Coding Rate

**DOI:** 10.3390/e26100818

**Published:** 2024-09-25

**Authors:** Zhengzhong Yi, Zhipeng Liang, Yulin Wu, Xuan Wang

**Affiliations:** Harbin Institute of Technology, Xili University Town, Nanshan District, Shenzhen 518055, China; zhengzhongyi@cs.hitsz.edu.cn (Z.Y.); liangzhipenghitsz@163.com (Z.L.); yulinwu@cs.hitsz.edu.cn (Y.W.)

**Keywords:** quantum error correction codes, quantum polar stabilizer codes, classical polar codes, coding rate, error-correcting capability, logical error rate

## Abstract

Inspired by classical polar codes, whose coding rate can asymptotically achieve the Shannon capacity, researchers are trying to find their analogs in the quantum information field, which are called quantum polar codes. However, no one has designed a quantum polar coding scheme that applies to quantum computing yet. There are two intuitions in previous research. The first is that directly converting classical polar coding circuits to quantum ones will produce the polarization phenomenon of a pure quantum channel, which has been proved in our previous work. The second is that based on this quantum polarization phenomenon, one can design a quantum polar coding scheme that applies to quantum computing. There are several previous work following the second intuition, none of which has been verified by experiments. In this paper, we follow the second intuition and propose a more reasonable quantum polar stabilizer code construction algorithm than any previous ones by using the theory of stabilizer codes. Unfortunately, simulation experiments show that even the stabilizer codes obtained from this more reasonable construction algorithm do not work, which implies that the second intuition leads to a dead end. Based on the analysis of why the second intuition does not work, we provide a possible future direction for designing quantum stabilizer codes with a high coding rate by borrowing the idea of classical polar codes. Following this direction, we find a class of quantum stabilizer codes with a coding rate of 0.5, which can correct two of the Pauli errors.

## 1. Introduction

Due to the great potential to solve certain problems that cannot be solved by classical computers with reasonable resources and time, quantum computing has attracted worldwide attention in the past twenty years. Nowadays, researchers have studied many physical systems that can be used to realize quantum computing, including superconducting circuits [1,2,3], trapped ions [4], and quantum dots [5]. However, limited by the hardware, none of these systems has realized reliable large-scale quantum computing, since qubits in these physical systems are easily influenced by the environment, which makes the state of qubits and hence the quantum information carried by them prone to error. Fortunately, quantum error-correcting (QEC) proposed by Shor and Steane [6,7] provides us with a solution to this problem.

Though quantum error-correcting codes (QECCs) provide us with a promising approach for fault-tolerant large-scale quantum computing, the high qubit overhead results from the low coding rate of most QECCs and challenges the current hardware level. Similar to classical error-correcting codes (CECCs), QECCs encode *n* (which is called code length) less reliable physical qubits (with error rate *p*) to obtain *k* more reliable logical qubits (with error rate $p_{L}<p$ after decoding and recovery). The ratio k/n is called the coding rate. Improving the coding rate is beneficial to reducing qubit overhead.

No matter whether for CECCs or QECCs, in general, we often increase the code length *n* to improve the reliability of the logical bits/qubits. Good CECCs have constant or even asymptotically increasing coding rates with the increase in code length *n* [8,9,10,11]. For instance, experimental results show that the coding rate of classical low-density parity check (CLDPC) codes [8,9,10] seems to achieve Shannon capacity. Another example is that Arikan has proved the coding rate of classical polar code [11] can asymptotically achieve the Shannon capacity.

These CECCs with high coding rates have inspired researchers to look for efficient QECCs. For example, inspired by CLDPCs, researchers proposed quantum low-density parity check (QLDPC) codes whose coding rate can remain constant [12,13,14,15,16,17,18,19,20]. However, the relationship between the coding rate of QLPDCs and quantum channel capacity is still not clear. If we take maximum single-letter coherent information (MSLCI), which is explained in Section 2.2, as quantum channel capacity, the asymptotic coding rate of certain QLDPCs seems to be rather lower than quantum channel capacity [21]. Another example of efficient QECCs research inspired by CECCs is quantum polar codes inspired by classical polar codes [11]. In previous work [22,23,24,25,26,27,28,29,30,31], some quantum polar coding schemes have been proposed. However, either they are based on the classical-quantum channel [22,23,24,26,28,29,31], or they ask for destructive measurements [25,27], which refers to the measurements that destruct the quantum information carried by quantum states. This makes them not applicable to quantum computing, where the computing channel is a pure quantum channel and destructive measurement should be avoided.

There are two intuitions in current research on quantum polar coding [22,23,24,25,26,27,28,29,30,31,32,33]. The first is that directly converting the classical polar coding circuits into quantum ones will lead to polarization phenomenon of quantum channels. The second is that based on the polarization phenomenon of quantum channels that arises from directly converting the classical polar coding circuits into quantum ones, one can design a quantum polar coding scheme for quantum computing. In our previous work [21], we proved the first intuition in two-dimensional-input quantum symmetric channels (QSCs). There are several previous works following the second intuition [22,25,27,32,33]. However, none of them are a quantum stabilizer code for quantum computing in a real sense, except the one in ref. [33]—either they are based on classical-quantum channels [22] or they ask for destructive measurement [25,27]. In ref. [33], the authors give a method to construct so-called “entanglement assisted quantum polar stabilizer codes”. However, there is no experiment to support the validity of these codes. The validity that we refer to is the error-correcting capability of the codes, which means one can decrease the logical error rate after error-correcting by increasing the code length, and a noise threshold can be observed. In this paper, we follow the second intuition and propose two construction algorithms, called the coherent-information-achieving (CA) algorithm and the block-selection (BS) algorithm, to construct quantum polar stabilizer codes (QPSCs). CA is the same as the construction in ref. [33], while we believe BS is more reasonable than CA because it is designed using the theory of stabilizer codes. By saying “using the theory of stabilizer codes”, we mean that the more reasonable construction has considered the weight of logical operators and stabilizers. Unfortunately, simulation experiments show that even the stabilizer codes obtained from this more reasonable construction algorithm, called the BS algorithm, do not work, which implies that the second intuition leads to a dead end.

Quantum stabilizer codes are an important class of QECCs. They avoid destructive measurements in error syndrome detection by measuring stabilizers. Different from the decoders of CECCs, which decode the value of the received bits into the value of bits before encoding, the decoders of quantum stabilizer codes decode the error syndromes which are composed of the measurement result of stabilizers into the errors that happen to each qubit. Quantum stabilizer codes can be represented by their stabilizer generators. Determining a stabilizer generator’s construction method is equivalent to determining a kind of quantum stabilizer code construction method. According to the code construction of classical polar code and the ranking of quantum coordinate channels, we propose two algorithms for constructing stabilizer generators of QPSCs. The first one ranks the quantum coordinate channels by the Bhattacharyya parameters. Since it allows the coding rate to achieve the channel capacity if we take MSLCI as quantum channel capacity, we name it the coherent-information-achieving (CA) construction algorithm. The second one ranks the quantum coordinate channels by the weight of their corresponding stabilizer generators. Since this algorithm ranks the coordinate channels in a block-by-block way, we name it the block-selection (BS) construction algorithm.

In classical polar codes, the frozen bits are frozen to a fixed value such as zero before encoding, which is a priori knowledge for decoders. In decoding, decoders will use this priori knowledge and decode all frozen bits into the fixed value. Hence, in the whole encoding-transmitting-decoding process, the frozen bits seem to suffer no error. This point is crucial to guarantee the reliability of polar codes, since the decoding of frozen bits will influence the decoding of information bits. For QPSCs, there are two kinds of assumptions about the frozen qubits—unreliable frozen qubits assumption and reliable frozen qubits assumption. The unreliable frozen qubits assumption assumes that the frozen qubits suffer from errors, as the information qubits do, which is the common assumption in the error correction of quantum computing, while the reliable frozen qubits assumption assumes that the frozen qubits do not suffer from errors, which might be too strong for quantum computing.

To test the error-correcting capability of QPSCs under these two construction algorithms, we perform simulation in a bit-flip channel under the above two assumptions, respectively. The simulation results show that, under both assumptions, for both CA and BS construction algorithms, in the range of *p* from $1\times 10^{-5}$ to $1\times 10^{-2}$, no evidence shows that the logical error rate (LER) of the single logical qubit $LER_{slq}$ can be decreased by increasing code length, and we cannot find the noise threshold. That is to say, these QPSCs do not work for quantum computing. Therefore, we come to a conclusion that the second intuition is too naive to guide us to design quantum polar coding scheme for quantum computing.

Based on the analysis of why QPSCs do not work, an alternative idea is proposed to construct stabilizer codes with high coding rate, which is to recursively expand the Tanner graph of certain stabilizer codes. Following this idea, we propose a class of stabilizer codes with a constant coding rate of 0.5, which can correct two of the Pauli errors.

The rest of this paper is organized as follows. Some preliminaries are introduced in Section 2, including stabilizer codes, quantum channel and its capacity, classical polar code and its code construction, and the polarization of two-dimensional-input quantum symmetric channels. In Section 3, we reveal the relationship between the channel quality of quantum coordinate channels and the weight of stabilizer generators and logical operators of QPSCs. In Section 4, we give the CA and BS construction algorithms of QPSCs. In Section 5, simulation results are given and analyzed. In Section 6, we provide a possible future direction of designing quantum stabilizers with high coding rates by borrowing the idea of classical polar codes. We conclude our work in Section 7.

## 2. Preliminaries

### 2.1. Stabilizer Codes

This section briefly introduces quantum stabilizer codes. Quantum stabilizer codes are an important class of QECCs, whose code construction is analogous to classical linear codes [34].

In quantum information theory, errors acting on a single qubit are generally modeled as elements of the Pauli group denoted by $G_{1}$, which consists of all Pauli operators together with multiplicative constants {±1,±i}, namely,

(1) $$G_{1}=\left\{\pm I,\pm iI,\pm X,\pm iX,\pm Y,\pm iY,\pm Z,\pm iZ\right\}$$

The general Pauli group $G_{n}$ on *n* qubits is the *n*-fold tensor product of $G_{1}$, namely, $G_{n}=G_{1}^{⊗n}$. Here, we use notation $w\left(\cdot \right)$ to denote the weight of an operator $E\in G_{n}$, namely, the number of qubits on which it acts nontrivially (with a Pauli operator other than the identity *I*). For instance, $w\left(X_{1}X_{2}I_{3}\right)=2$.

A quantum stabilizer code *C* encodes *k* logical qubits into *n* physical qubits. Denote the code space by $Q_{C}$ which is a subspace of ${\left(C^{2}\right)}^{⊗n}$. The code space $Q_{C}$ is stabilized by a stabilizer group 𝒮 which is a subgroup of $G_{n}$. Giving the stabilizer group 𝒮 of a code C is equivalent to explicitly giving its code space $Q_{C}$. Using the stabilizer group 𝒮, the code space $Q_{C}$ can be defined as

(2) $$\begin{array}{c} Q_{C}=\left\{|\psi \rangle \in {\left(C^{2}\right)}^{⊗n}:\;S|\psi \rangle =|\psi \rangle ,\forall \;S\in 𝒮\right\} \end{array}$$

A group *G* can be generated by a set of independent elements $g_{1},\cdots ,g_{l}$, which means each element in *G* can be written as a product of elements from the list $g_{1},\cdots ,g_{l}$. We say $g_{1},\cdots ,g_{l}$ are generators of group *G*, and we write $G=\langle g_{1},\cdots ,g_{l}\rangle$.

Similar to classical linear codes, a stabilizer code *C* can be expressed as a 3-tuple $\left[n,k,d\right]$, which means it encodes *k* logical qubits into *n* physical qubits and its code distance is *d*. For an $\left[n,k,d\right]$ code *C*, its stabilizer group 𝒮 has n−k independent generators, namely, $𝒮=\langle S_{1},\cdots ,S_{n-k}\rangle$. Besides, we can also find another subgroup L of $G_{n}$ that has *k* pairs of generators, namely, $L=\langle {\bar{X}}_{1},{\bar{Z}}_{1},\cdots ,{\bar{X}}_{k},{\bar{Z}}_{k}\rangle$, whose elements commute with all elements in 𝒮 [35]. The elements in group L are called the logical operators of code *C*. Code distance *d* is defined as the minimum weight of the logical operators, namely,

(3) $$\begin{array}{c} d=\min_{P\in L}w(P) \end{array}\;$$

For a $\left[n,k\right]$ stabilizer code C, its encoding process can be depicted by a unitary operator $U_{enc}$ acting on *n* qubits. These *n* qubits can be divided into two different sets. The first set consists of *kdata qubits* (or called logical qubits) which contains information to be encoded, and the second set consists of n−k *ancilla qubits* which are set to a fixed state, such as ${|0\rangle }^{⊗n-k}$. The Pauli operators acting on *k* logical qubits before encoding will be transformed to *k* sets of logical operators by the encoding operator $U_{enc}$ after encoding, namely, ${\bar{L}}_{i}=U_{enc}P_{i}U_{enc}^{†}$, 1≤i≤k. The Pauli operators acting on n−k ancilla qubits are transformed to stabilizer generators by the encoding operator $U_{enc}$ after encoding, namely, $S_{i}=U_{enc}P_{i}U_{enc}^{†}$, k+1≤i≤n.

### 2.2. Pure Quantum Channel and Its Capacity

#### 2.2.1. Pure Quantum Channel

If the inputs and outputs of a channel are both arbitrary quantum states, it is a pure quantum channel. Different from it, if the channel input or output is restricted to some sets of quantum states, not arbitrary states, it is not a pure quantum channel. A typical instance is the classical-quantum channel [22,23,24,26,28,29,31], whose input is classical variables or quantum states corresponds to the sets of value that these variables can take. The behavior of a quantum channel E acting on a quantum system *P* can be described by a set of operator elements ${\left\{E_{i}\right\}}_{i=1,\cdots n}$, which satisfies $\sum_{i=1}^{n}E_{i}^{†}E_{i}=I$ [34]. Suppose the state of quantum system *P* is $\rho^{P}$, and *P* is subject to the quantum channel E, which changes system *P* to $P^{\prime }$ and maps the state $\rho^{P}$ to $\rho^{P^{\prime }}$. Using operator-sum representation [34], the transformation of state $\rho^{P}$ can be described as

(4) $$\begin{array}{c} \rho^{P^{\prime }}=E\left(\rho^{P}\right)=\sum_{i=1}^{n}E_{i}\rho^{P}E_{i}^{†} \end{array}\;$$

Common quantum channels include bit-flip channel, phase-flip channel and depolarizing channel, whose operator elements are $\left\{\sqrt{p}X,\sqrt{1-p}I\right\}$, $\left\{\sqrt{p}Z,\sqrt{1-p}I\right\}$, and $\left\{\sqrt{p/3}X,\sqrt{p/3}Y,\sqrt{p/3}Z,\sqrt{1-p}I\right\}$, respectively.

In our previous work [21], we propose a mathematical tool, **basis transition probability matrix (BTPM)**, which is inspired by the transition probability matrix (TPM) of classical channels, to depict the behavior of quantum channels. Besides, we prove that, for a quantum channel E having BTPM, giving its BTPM is equivalent to giving its operator elements ${\left\{E_{i}\right\}}_{i=1,\cdots n}$, which satisfies $\sum_{i=1}^{n}E_{i}^{†}E_{i}=I$. We take the bit flip channel and two-dimensional-input Pauli channel as examples and choose the computational basis {|0〉,|1〉} to be the basis of their input space. For the bit flip channel that flips |0〉 and |1〉 with the same probability *p*, its BTPM is

(5) $$\begin{array}{c} \;|0\rangle \;|1\rangle \\ \begin{array}{c} |0\rangle \\ |1\rangle \end{array}\left(\begin{array}{cc} 1-p & p\\ p & 1-p \end{array}\right) \end{array}$$

And for the two-dimensional-input Pauli channel, which has four operation elements $\{\sqrt{p_{1}}I,$$\sqrt{p_{2}}X,\sqrt{p_{3}}Y,\sqrt{p_{4}}Z\}\;\left(\sum_{i=1}^{4}p_{i}=1\right)$, its BTPM is

(6) $$\begin{array}{c} \;|0\rangle \;|1\rangle \\ \begin{array}{c} |0\rangle \\ |1\rangle \end{array}\left(\begin{array}{cc} p_{1}+p_{4} & p_{2}+p_{3}\\ p_{2}+p_{3} & p_{1}+p_{4} \end{array}\right) \end{array}$$

In [21], we also define two classes of quantum channel, **quantum symmetric channel (QSC)** and **quantum quasi symmetric channel (QQSC)**. For a quantum channel that has BTPM, if each row of the BTPM is a permutation of the first row, then this quantum channel is symmetric with respect to its input. If each column of the BTPM is a permutation of the first column, then this quantum channel is symmetric with respect to its output. If a quantum channel is symmetric with respect to both its input and output, then this channel is called QSC. If a channel is symmetric with respect to its input but might not to its output, and its BTPM can be divided into several submatrices by column, each of which satisfies that each column of it is a permutation of the first column of it, then this channel is called a QQSC.

#### 2.2.2. Quantum Channel Capacity

Coherent information of a quantum channel E, denoted by $I(\rho^{P},E)$, is the quantity measuring the amount of quantum information transmitted in the channel [36], where $\rho^{P}$ is the state of quantum system *P* before being transformed by E. Coherent information is believed to be the analog of classical mutual information in quantum information theory [34] and a quantity to measure the channel capacity of pure quantum channels [37,38,39,40,41,42,43,44,45,46]. However, since the coherent information has superadditivity, it is still not clear how to calculate quantum channel capacity precisely by coherent information. Here, we use maximum single letter coherent information (MSLCI) [21] to measure quantum channel capacity, which is believed to be a lower bound of the average coherent information of multiple uses of a quantum channel [47].

For a classical symmetric channel, Arikan has proved that its symmetric capacity is its Shannon capacity. Similarly, in [21] we prove that the MSLCI of a two-dimensional-input QQSC equals its symmetric coherent information, whose definition is as follows.

 **Definition 1** **(Symmetric coherent information). ** *For a quantum channel E, the number of whose input qubits is n, its input state can be represented by $\rho =\sum_{i=1}^{2^{n}}q_{i}|i\rangle \langle i|$, its symmetric information $I_{U}$ is defined as the coherent information I(ρ,E) when $q_{1}=\cdots =q_{2^{n}}=\frac{1}{2^{n}}$, namely,*

(7) $$I_{U}\equiv I\left(\rho =\sum_{i=1}^{2^{n}}\frac{1}{2^{n}}|i\rangle \langle i|,E\right)$$

### 2.3. Classical Polar Code and Its Code Construction

For a binary-input discrete memoryless channel (B-DMC) W:X→Y with input alphabet X, output alphabet Y and transition probabilities $W\left(y|x\right)$, x∈X={0,1}, y∈Y, Arikan defines two parameters [11], the symmetric capacity and the Bhattacharyya parameter, to measure its quality. The symmetric capacity of a B-DMC is defined as

(8) $$I\left(W\right)≜\sum_{y\in Y}\sum_{x\in X}\frac{1}{2}W\left(y|x\right)\log\frac{W(y|x)}{\frac{1}{2}W\left(y|0\right)+\frac{1}{2}W\left(y|1\right)}$$

and its Bhattacharyya parameter is defined as

(9) $$Z\left(W\right)≜\sum_{y\in Y}\sqrt{W(y|0)W(y|1)}$$

Arikan has proved that the symmetric capacity I(W) is equal to the Shannon capacity when *W* is a symmetric channel. One can see Z(W) takes values in $\left[0,1\right]$, and I(W) also take values in $\left[0,1\right]$ if we use base-2 logarithms. When I(W) tends to 1, Z(W) tends to 0, and when I(W) tends to 0, Z(W) tends to 1.

Classical polar coding proposed by Arikan [11] is the only coding scheme whose coding rate is proved to achieve the symmetric capacity of any given B-DMC. This scheme is based on channel polarization, which consists of two processes, channel combining and channel splitting.

**Figure 1:**
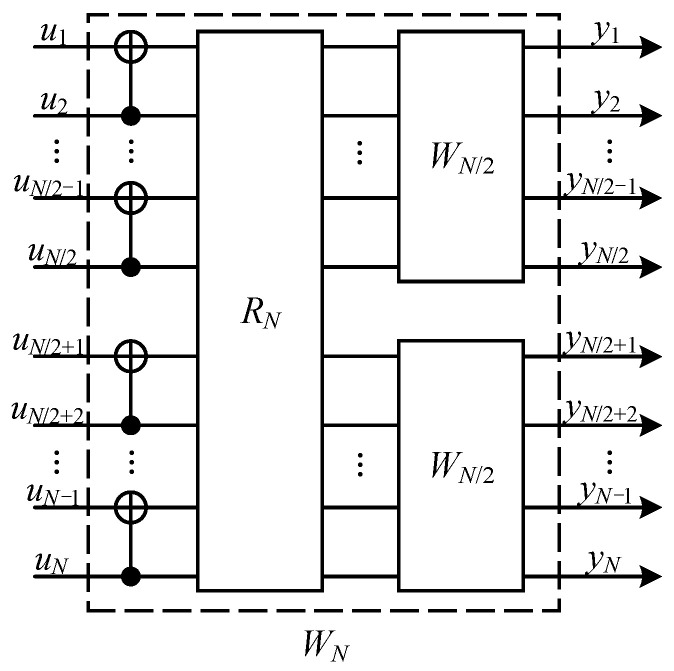
Classical channel polarization. $R_{N}$ is the reverse shuffle operation. Two independent copies of $W_{N/2}$ are combined to produce the channel $W_{N}$. Please see ref. [11] for more detail.

As shown in Figure 1, in channel combining, we combine *N* copies of a given B-DMC W:X→Y (whose symmetric capacity is I(W)) with input alphabet X and output alphabet Y in a recursive manner and obtain a combined channel $W_{N}:X^{N}\to Y^{N}$ ($X^{N}$ and $Y^{N}$ are the *N*-power extension alphabet of X and Y, respectively) which maps the vector $\left(x_{1},\cdots ,x_{N}\right)$ to $\left(y_{1},\cdots ,y_{N}\right)$. Here, we will use $x_{1}^{N}$ as a shorthand for vector $\left(x_{1},\cdots ,x_{N}\right)$. In channel splitting, the combined channel $W_{N}$ is split back into a set of *N* binary-input coordinate channels ${\left\{W_{N}^{\left(i\right)}\right\}}_{1\leq i\leq N}$. The coordinate channel $W_{N}^{\left(i\right)}$ is define as

(10) $$W_{N}^{\left(i\right)}:X\to Y^{N}\times X^{i-1},1\leq i\leq N$$

As *N* becomes large, the symmetric capacity of N×I(W) coordinate channels will asymptotically tend to 1 and that of the rest $N\times (1-I\left(W\right))$ coordinate channels will asymptotically tend to 0. It means N×I(W) coordinate channels are noiseless, which can be used to perfectly transmit information bits, while the rest $N\times (1-I\left(W\right))$ coordinate channels are completely noisy and can be used to transmit frozen bits. After channel polarization, we compute the channel quality of each coordinate channel $W_{N}^{\left(i\right)}$, and rank the coordinate channels in descending order according to their channel quality. Then, we choose the former $\left\lfloor N\times I(W)\right\rfloor (\left\lfloor \cdot \right\rfloor$ means the value is rounded down) coordinate channels to transmit information bits, and the rest to transmit frozen bits.

Arikan has proved that [11] if the primal channel *W* is a binary erasure channel (BEC), the channel quality of classical coordinate channels $\left\{W_{N}^{\left(i\right)}\right\}$ can be measured by the Bhattacharyya parameter, and they can be precisely computed through the following recursion:

(11) $$\begin{array}{cc} Z\left(W_{N}^{\left(2j-1\right)}\right) & =2Z\left(W_{N/2}^{\left(j\right)}\right)-Z{\left(W_{N/2}^{\left(j\right)}\right)}^{2}\\ Z\left(W_{N}^{\left(2j\right)}\right) & =Z{\left(W_{N/2}^{\left(j\right)}\right)}^{2} \end{array}$$

However, if the primal channel *W* is a binary symmetric channel (BSC), there is no such recursion to precisely compute the channel quality of classical coordinate channels $\left\{W_{N}^{\left(i\right)}\right\}$, only the following relationships:

(12) $$\begin{array}{cc} Z\left(W_{2N}^{\left(2i-1\right)}\right) & \leq 2Z\left(W_{N}^{\left(i\right)}\right)-Z{\left(W_{N}^{\left(i\right)}\right)}^{2}\\ Z\left(W_{2N}^{\left(2i\right)}\right) & =Z{\left(W_{N}^{\left(i\right)}\right)}^{2} \end{array}$$

Since the TPM of $W_{N}^{\left(i\right)}$ is exponential to the code length *N*, precisely computing the Bhattacharyya parameter of these $\left\{W_{N}^{\left(i\right)}\right\}$ is intractable. Fortunately, ref. [48] has proved that, if the primal channel *W* is a BSC, we can still use Equation (11) to approximately estimate the Bhattacharyya parameter of classical coordinate channels $\left\{W_{N}^{\left(i\right)}\right\}$.

### 2.4. Polarization of Two-Dimensional-Input Quantum Symmetric Channels

For a two-dimensional-input quantum channel that has a BTPM, except for its symmetric coherent information, we can also use the Bhattacharyya parameter to measure its channel quality, which is defined as

(13) $$Z\left(E\right)≜\sum_{|i\rangle \in B_{\mathrm{out}\;}}\sqrt{\mathrm{Pr}(|i\rangle ||0\rangle )\mathrm{Pr}(|i\rangle ||1\rangle )}$$

where $B_{\mathrm{out}\;}$ is the basis of output space of E, $B_{\mathrm{in}}=\left\{|0\rangle ,|1\rangle \right\}$ is the basis of input space of E, and Pr(|i〉||k〉), $|k\rangle \in B_{\mathrm{in}\;}$,$|i\rangle \in B_{\mathrm{out}\;}$ are basis transition probabilities.

#### Polarization of Channel Quality of Quantum Coordinate Channels

**Figure 2:**
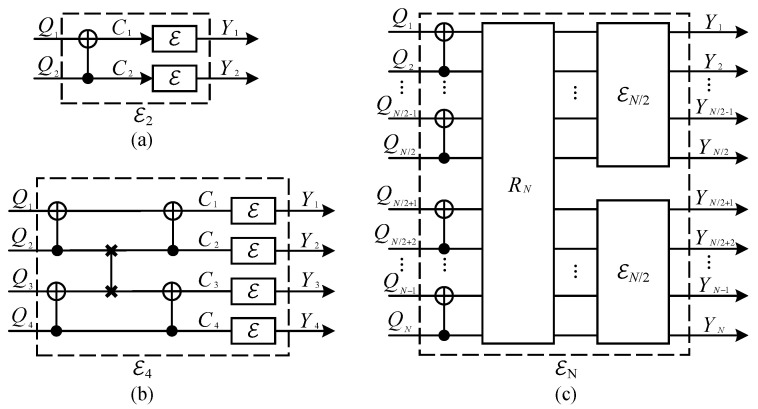
Quantum channel polarization circuits: (**a**) Two primal channel E combines to form channel $E_{2}$. (**b**) Two $E_{2}$ combines to form channel $E_{4}$. (**c**) Two $E_{N/2}$ combines to form channel $E_{N}$, $R_{N}$ is the reverse shuffle operator [21].

As shown in Figure 2, quantum channel polarization is similar to classical channel polarization and also consists of two processes—quantum channel combining and quantum channel splitting [21]. We only briefly introduce some concepts and conclusions here.

Through quantum channel combining, one can combine *N* copies of primal quantum channel $E:\rho^{Q}\to \rho^{Y}$ in a recursive way and obtain a quantum combined channel $E_{N}:\rho^{Q_{1}\cdots Q_{N}}\to \rho^{Y_{1}\cdots Y_{N}}$. The difference is that we replace the XOR gates in classical channel combining by quantum CNOT gates, and use quantum SWAP gates to realize the reverse shuffle operator. One can see that this process is realized by converting the classical polar coding circuits to the quantum version. In the quantum channel splitting process, we split the quantum combined channel $E_{N}$ back into *N* quantum coordinate channels $E_{N}^{\left(i\right)}:\;\rho^{Q_{i}}\to \rho^{Y_{1}\cdots Y_{N},R_{1}\cdots R_{i-1}}$, where $R_{i}$ is the reference system of $Q_{i}$, 1≤i≤N. Here, we use notation $\rho^{Q_{1}^{N}}$ to denote $\rho^{Q_{1}\cdots Q_{N}}$ which is similar to Arikan’s shorthand for a vector. Hence the quantum combined channel and quantum coordinate channel can be rewritten as $E_{N}:\rho^{Q_{1}^{N}}\to \rho^{Y_{1}^{N}}$ and $E_{N}^{\left(i\right)}:\;\rho^{Q_{i}}\to \rho^{Y_{1}^{N}R_{1}^{i-1}}$, respectively.

It has been proved that if the primal quantum channel E is a two-dimensional-input QSC with two-dimensional output, the quantum combined channel $E_{N}$ is a QSC, and the quantum coordinate channels $\left\{E_{N}^{\left(i\right)}\right\}$ are all two-dimensional-input QQSCs [21]. Besides, if the BTPM of the primal QSC E and the TPM of classical primal BSC *W* are the same, the MSLCI $I(\rho^{Q_{i}},E_{N}^{\left(i\right)})$ of the quantum coordinate channel $E_{N}^{\left(i\right)}$ equals to the Shannon capacity $I(W_{N}^{\left(i\right)})$ of the classical coordinate channel $W_{N}^{\left(i\right)}$. Since classical coordinate channels $\left\{W_{N}^{\left(i\right)}\right\}$ polarize, quantum coordinate channels $\left\{E_{N}^{\left(i\right)}\right\}$ polarize as well.

In this paper, the quantum channel E that we consider is a bit-flip channel, namely, only Pauli *X* error happens. The reason for this is that correcting Pauli *X* error is the prerequisite to correct arbitrary Pauli errors. If a quantum stabilizer code does not work in a bit-flip channel, then it will not work in a channel where arbitrary Pauli errors might happen.

The BTPM of a bit-flip channel is the same as the TPM of BSC *W*. Hence, we have $I\left(\rho^{Q_{i}},E_{N}^{\left(i\right)}\right)=\;I\left(W_{N}^{\left(i\right)}\right)$ and $Z\left(E_{N}^{\left(i\right)}\right)={Z(W}_{N}^{\left(i\right)})$. Same as for the approximation method of the Bhattacharyya parameter of classical channels mentioned in Section 2.3, we can approximately estimate the Bhattacharyya parameter of quantum coordinate channels $\left\{E_{N}^{\left(i\right)}\right\}$ by the following recursion,

(14) $$\begin{array}{cc} Z\left(E_{N}^{\left(2i-1\right)}\right) & =2Z\left(E_{N/2}^{\left(i\right)}\right)-{\left(Z\left(E_{N/2}^{\left(i\right)}\right)\right)}^{2}\\ Z\left(E_{N}^{\left(2i\right)}\right) & ={\left(Z\left(E_{N/2}^{\left(i\right)}\right)\right)}^{2} \end{array}$$

The greater $Z(E_{N}^{\left(i\right)})$ is, the less reliable $E_{N}^{\left(i\right)}$ is.

## 3. The Weight of Stabilizer Generators and Logical Operators of Quantum Polar Stabilizer Codes

This section reveals the relationship between the channel quality of quantum coordinate channels and the weight of their corresponding stabilizer generators and logical operators.

According to the intuition that one can design a quantum polar coding scheme for quantum computing based on the polarization phenomenon of quantum channels that arise from directly converting the classical polar coding circuits into quantum ones, the circuits shown in Figure 2 might correspond to a QPSC encoding circuit.

**Figure 3:**
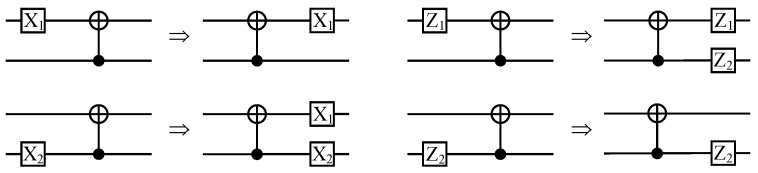
Transformation of Pauli *X* operators and Pauli *Z* operators under conjugation by CNOT gate.

As shown in Figure 2, the process $|Q_{1}^{N}\rangle \to |C_{1}^{N}\rangle$ can be seen as a QPSC encoding circuit denoted by $E_{N}$, and this encoding circuit can be specified as a unitary operator $U_{enc}$. As we mention in Section 2.1, the Pauli *Z* operators (only the Z-type operators need to be considered since only Pauli *X* errors are considered) acting on the frozen qubits are transformed into stabilizer generators, while the Pauli operators acting on the data qubits are transformed into logical operators by $U_{enc}$. We denote the unitary operator of CNOT gate by notation $U_{CNOT}$, with qubit 1 as the target and qubit 2 as the control. Figure 3 shows that if a Pauli *Z* operator (whose weight is 1) “passes through” the target position of a CNOT gate, its weight becomes 2, while if it “passes through” the control position, its weight remains invariant. The transformation of a Pauli *X* operator is right the opposite.

To reveal the relationship between the weight of stabilizer generators and logical operators of QPSCs and the channel quality of quantum coordinate channels, we first define two concepts “node” and “path” for the quantum polarization circuits.

 **Definition 2** **(Node).**  *The operation which acts on a single qubit in a CNOT gate is defined to be a node. We use notation* ⨁ *to denote the target node which represents “XOR” operation of a CNOT gate, and notation* ⨀ *to denote the control node which represents the identity operation of a CNOT gate.*

It is obvious that the encoding circuit $E_{N}$ consists of $n=\log_{2}N$ CNOT gate blocks, and each block contains N/2 CNOT gates (or equivalently *N* nodes). Arbitrary two adjacent CNOT gate blocks are connected by reverse shuffle operation realized by SWAP gates. The index of CNOT gate block is numbered from right to left, that is to say, the index of the rightmost CNOT gate block is 1, and that of the leftmost one is *n*. The index of a node located at a CNOT gate block is numbered from top to bottom. One can see that if the index of a node is an odd number, it must be a target node ⨁, if that of a node is an even number, it must be a control node ⨀.

 **Definition 3** **(Path of a qubit passing through an encoding circuit).**  *A path $P_{j}$ of the jth input qubit $Q_{j}$ in the encoding circuit $E_{N}$ with length N is defined as an ordered set $\left\{{node}_{n}^{j},\cdots ,{node}_{1}^{j}\right\}$, where ${node}_{i}^{j}\in \left\{⨁,⨀\right\}$, 1≤i≤n, 1≤j≤N.*

It is obvious that each ordered set which represents a complete path from the input of $E_{N}$ to the output contains $n=\log_{2}N$ nodes. Here, the reason for labeling the index *i* of node from n to 1 rather than 1 to n is to keep this index consistent with the index of the CNOT gate blocks (i.e., ${node}_{i}^{j}$ is located at the *i*th CNOT gate block).

Let us take the encoding circuit $E_{4}$ for example. As shown in Figure 2b, the path of the first qubit is $P_{1}=\left\{⊕,⊕\right\}$, the second is $P_{2}=\left\{⊙,⊕\right\}$, the third is $P_{3}=\left\{⊕,⊙\right\}$, and the fourth is $P_{4}=\left\{⊙,⊙\right\}$.

For path $P_{j}$ in $E_{N}$, we observe that $P_{j}$ corresponds to not only the *j*th stabilizer generator $S_{j}$, which comes from the transformation of Pauli *Z* operator acting on qubit $Q_{j}$, but also the *j*th quantum coordinate channel $E_{N}^{\left(j\right)}$, whose input is $Q_{j}$.

 **Definition 4** **(Length of a path).**  *For a path $P=\left\{{node}_{n},\cdots ,{node}_{1}\right\}$, its length is defined as the number of nodes that it contains, denoted by $\left|P\right|$.*

 **Definition 5** **(Subpath).**  *For a path $P=\left\{{node}_{n},\cdots ,{node}_{1}\right\}$ and a path $P^{\prime }=\left\{{node}_{k},{node}_{k-1},\right.$$\left.\cdots ,{node}_{k-r}\right\}$, if $P^{\prime }$ is part of P, namely, $P=\left\{{node}_{n},\cdots ,P^{\prime },\cdots ,{node}_{1}\right\}=\left\{{node}_{n},\cdots ,\right.$$\left.{node}_{k},{node}_{k-1}\cdots ,{node}_{k-r},\cdots ,{node}_{1}\right\}$, $P^{\prime }$ is a subpath of path P, denoted by $P^{\prime }\in P$. We notice that $P^{\prime }$ follows ${node}_{k+1}$ and is followed by ${node}_{k-r-1}$. Specially, if k=n, k−r>1, we call $P^{\prime }$ the **former path*** *of P, if k<n, k−r=1, we call $P^{\prime }$ the* ***latter path*** *of P, and if k=n, k−r=1, we say $P^{\prime }$ is identical to P, denoted by $P^{\prime }=P$.*

For arbitrary two paths $P_{i}$ and $P_{j}$, as long as they have one node not the same, we say they are different, denoted by $P_{i}\neq P_{j}$. It is obvious that two paths with different lengths must be different.

 **Lemma 1** **(Uniqueness of path).**  *Arbitrary two paths $P_{i}$ and $P_{j}$(1≤i≠j≤N) in the same encoding circuit $E_{N}$ are different, namely, $P_{i}\neq P_{j}$. That is to say, each path in $E_{N}$ is unique.*

 **Proof.**  Here, we apply the mathematical induction to prove it.

 **Step 1:**  For n=1, as shown in Figure 2a, the encoding circuit $E_{2}$ only contains one CNOT gate, it is obvious these two paths are different.
 **Step 2:**  For n=k(k>1), we assume that arbitrary two paths in encoding circuit $E_{N/2}\;\left(N/2=2^{k}\right)$ are different.
 **Step 3:**  For n=k+1(k>1), two independent copies of $E_{N/2}$ are combined to produce a larger encoding circuit $E_{N}$ in a recursive way. As shown in Figure 2c, we call the upper encoding circuit $E_{N/2}$ block 1, and the lower encoding circuit $E_{N/2}$ block 2. There are 3 cases in total that we should consider.
 **Case 1:**  For arbitrary two paths $P_{1}^{\prime }$ and $P_{2}^{\prime }$ both in block 1 or block 2, according to step 2, they are different, so we cannot find two identical paths $P_{1}$ and $P_{2}$ in $E_{N}$, which satisfies $P_{1}^{\prime }\in P_{1}$ and $P_{2}^{\prime }\in P_{2}$.
 **Case 2:**  For two paths $P_{1}^{\prime }$ in block 1 and $P_{2}^{\prime }$ in block 2, if they are different, we also cannot find two identical paths $P_{1}$ and $P_{2}$ in $E_{N}$, which satisfies $P_{1}^{\prime }\in P_{1}$ and $P_{2}^{\prime }\in P_{2}$.
 **Case 3:**  For two paths $P_{1}^{\prime }$ in block 1 and $P_{2}^{\prime }$ in block 2, if they are identical, according to the recursive construction of $E_{N}$ from two copies of $E_{N/2}$, we can see that path $P_{1}^{\prime }$ follows a target node ⊕, and path $P_{2}^{\prime }$ follows a control node ⊙. These target node ⊕ and control node ⊙ belong to the same CNOT gate. Hence, we have two different paths $P_{1}=\left\{⊕,P_{1}^{\prime }\right\}$ and $P_{1}=\left\{⊙,P_{2}^{\prime }\right\}$.

Therefore, no matter in which case, we cannot find two identical paths in the same encoding block $E_{N}$, which completes the proof.    □

 **Lemma 2** **(Relation between two paths which have only one different node).**  *For two paths $P_{i}=\left\{{node}_{n}^{i},\cdots ,{node}_{1}^{i}\right\}$ and $P_{j}=\left\{{node}_{n}^{j},\cdots ,{node}_{1}^{j}\right\}\;\left(i<j\right)$ in encoding circuit $E_{N}$, the number of target node* ⊕ *contained by $P_{i}$ is $n_{⊕}^{i}$ and that contained by $P_{j}$ is $n_{⊕}^{j}$. We have $n_{⊕}^{i}=n_{⊕}^{j}+1$ if and only if these two paths pass through the same CNOT gate.*

 **Proof.**  (1) Necessity: Assuming that the *m*th ($2\leq m\leq log_{2}\left(n-1\right)$) nodes of $P_{i}$ and $P_{j}$ belong to the same CNOT gate, namely, $P_{i}=\left\{P_{i}^{for},⊕,P_{i}^{lat}\right\}$ and $P_{j}=\left\{P_{j}^{for},⊙,P_{j}^{lat}\right\}$, where $P_{i}^{for}$ and $P_{j}^{for}$ are the former path of $P_{i}$ and $P_{j}$ respectively, and $P_{i}^{lat}$ and $P_{j}^{lat}$ are the latter path of $P_{i}$ and $P_{j}$, respectively.

Since the *m*th nodes of $P_{i}$ and $P_{j}$ belong to the same CNOT gate, according to the recursive construction of the encoding circuit, we must have $P_{i}^{lat}=P_{j}^{lat}$.

Suppose the indexes of ⊕ and ⊙ are *a* and *b*, respectively, since they belong to the same CNOT gate, we have b=a+1. According to the recursive construction of encoding circuit, the node ⊕ with index *a* located at the (m+1)th CNOT gate block of $E_{N}$ must following ${node}_{m+2}$ with index 2a (or 2a−1) located at the (m+2)th CNOT gate block, and node ⊙ with index b=a+1 located at the (m+1)th CNOT gate block must following ${node}_{m+2}^{\prime }$ with index 2b (or 2b−1) located at the (m+2)th CNOT gate block, which means the index of ${node}_{m+2}$ and ${node}_{m+2}^{\prime }$ must both be odd or even numbers. If they are both even numbers, these two nodes are both control nodes, and if they are both odd numbers, these two nodes are both target nodes. Recursively using this relation, we have $P_{i}^{for}=P_{j}^{for}$; thus, $n_{⊕}^{i}=n_{⊕}^{j}+1$.

(2) Sufficiency: Using proof by contradiction, we assume that there exists a $path_{h}$, which satisfies $n_{⊕}^{h}=n_{⊕}^{j}+1$, but $path_{h}$ and $path_{j}$ do not pass through the same CNOT gate. Suppose that the *l*th nodes of $path_{h}$ and $path_{j}$ are different, according to the necessity above, at the CNOT gates block corresponding to the *l*th node, there is a path $path_{i}$ that passes through the same CNOT gate as $path_{j}$, which satisfies $n_{⊕}^{i}=n_{⊕}^{j}+1$, and their former paths and latter paths are the same. Thus, we have $path_{h}=path_{i}$, which contradicts Lemma 1. Thus, the path $path_{h}$ above does not exist.

The proof is completed.    □

 **Lemma 3** **(The weight of stabilizer generator and logical operator).**  *For the ith path $P_{i}=\left\{{node}_{n}^{i},\cdots ,{node}_{1}^{i}\right\}$ in encoding circuit $E_{N}$, the number of target node* ⊕ *that it contains is denoted by $n_{⊕}^{i}$ and that of control node* ⊙ *is denoted by $n_{⊙}^{i}$ (we can see that $n_{⊕}^{i}+n_{⊙}^{i}=\left|P\right|=n=\log_{2}N$). If the ith input qubit $Q_{i}$ of $E_{N}$ is a frozen qubit and its state is set to |0〉, the weight of the ith stabilizer generator (Z-type) $S_{i}$ is $2^{n_{⊕}^{i}}$. Likewise, if it is logical qubit, the weight of ith logical X operator $\bar{X_{i}}$ is $2^{n_{⊙}^{i}}$.*

 **Proof.**  Since, as mentioned in Section 2.1, the behavior of $E_{N}$ can be specified as a unitary operator $U_{E_{N}}$. If the *i*th input qubit $Q_{i}$ of $E_{N}$ is set to state |0〉, it is a frozen qubit, and the Pauli *Z* operator $Z_{i}$ acting on $Q_{i}$ is transformed to the *i*th stabilizer generators $S_{i}$ under conjugation by $U_{E_{N}}$, namely, $S_{i}=U_{E_{N}}Z_{i}U_{E_{N}}^{†}$. If $Q_{i}$ is a logical qubit, the Pauli *X* operator acting on it are transformed to logical *X* operator under conjugation by $U_{E_{N}}$, namely, $\bar{X_{i}}=U_{E_{N}}X_{i}U_{E_{N}}^{†}$.

If $Q_{i}$ is a frozen qubit, we consider how the Pauli *Z* operator $Z_{i}$ transforms under conjugation by $U_{E_{N}}$. As shown in Figure 3, if a Pauli *Z* operator (whose weight is 1) passes through a target node ⊕ of a CNOT gate, its weight becomes 2, while if it passes through a control node ⊙, its weight remains invariant. If we assume that ${node}_{n}^{i}$ of $P_{i}$ is target node ⊕, $Z_{i}$ become $Z_{i}Z_{i-1}$ under conjugation by the first CNOT gate. After the conjugation, $Z_{i}$ continues along a subpath $P_{i}^{\prime }\in P_{i}$, while $Z_{i-1}$ along another path $P_{i-1}^{\prime }$. However, as we prove in Lemma 1, we must have $P_{i}^{\prime }=P_{i-1}^{\prime }$, since $P_{i-1}^{\prime }$ follows a control node ⊙ while $P_{i}^{\prime }$ follows a target node ⊕, and these two nodes belong to a same CNOT gate. Thus, the transformation of weight of $Z_{i-1}$ along $P_{i-1}^{\prime }$ is the same as that of $Z_{i}$ along $P_{i}^{\prime }$, which simultaneously multiplies by 2 or remains invariant dependent on the first node of $P_{i}^{\prime }$ is ⊕ or ⊙. Recursively using this law, the weight of the *i*th stabilizer generators $S_{i}$ must be $2^{n_{⊕}^{i}}$.

Likewise, if $Q_{i}$ is a logical qubit, the analysis process is the same and the weight of the *i*th logical *X* operator $\bar{X_{i}}$ is $2^{n_{⊙}^{i}}$.    □

 **Theorem 1** **(Negative correlation between the weight of stabilizer generators and the channel quality of quantum coordinate channels).**  *For two paths $P_{a}=\left\{{node}_{n}^{a},\cdots ,{node}_{1}^{a}\right\}$ and $P_{b}=\left\{{node}_{n}^{b},\cdots ,{node}_{1}^{b}\right\}$ in encoding circuit $E_{N}$, the number of target node* ⊕ *contained by $P_{a}$ is $n_{⊕}^{a}$ and that contained by $P_{b}$ is $n_{⊕}^{b}$. If $n_{⊕}^{a}>n_{⊕}^{b}$, the Bhattacharyya parameter of quantum coordinate channel $E_{N}^{\left(a\right)}$ is greater than that of $E_{N}^{\left(b\right)}$, namely, $Z\left(E_{N}^{\left(a\right)}\right)>Z\left(E_{N}^{\left(b\right)}\right)$.*

 **Proof.**  Since $n_{⊕}^{a}>n_{⊕}^{b}$, we can suppose $n_{⊕}^{a}=n_{⊕}^{b}+x$. Here, we only consider the case that x=1 (i.e., $n_{⊕}^{a}=n_{⊕}^{b}+1$). If in this case we can prove $Z\left(E_{N}^{\left(a\right)}\right)>Z\left(E_{N}^{\left(b\right)}\right)$, this relation holds for arbitrary x≥1.

By Lemma 2, when $n_{⊕}^{a}=n_{⊕}^{b}+1$, $P_{a}$ and $P_{b}$ can be rewritten as $P_{a}=\left\{P_{a}^{for},⊕,P_{a}^{lat}\right\}$ and $P_{b}=\left\{P_{b}^{for},⊙,P_{b}^{lat}\right\}$, where $P_{a}^{for}=P_{b}^{for}$, $P_{a}^{lat}=P_{b}^{lat}$, and the two nodes ⊕ and ⊙ belong to a same CNOT gate. Suppose this CNOT gate is located at the $\left(m+1\right)$th CNOT gates block and the index of nodes ⊕ located at this CNOT gates block is $i^{\prime }$, the index of nodes ⊙ must be $j^{\prime }=i^{\prime }+1$. According to Equation (14), we have,

(15) $$\begin{array}{c} \begin{array}{c} Z\left(E_{\frac{N}{2^{n-m-1}}}^{\left(i^{\prime }\right)}\right)=2Z\left(E_{\frac{N}{2^{n-m}}}^{\left(i^{\prime \prime }\right)}\right)-{\left(Z\left(E_{\frac{N}{2^{n-m}}}^{\left(i^{\prime \prime }\right)}\right)\right)}^{2}\\ Z\left(E_{\frac{N}{2^{n-m-1}}}^{\left(j^{\prime }\right)}\right)={\left(Z\left(E_{\frac{N}{2^{n-m}}}^{\left(i^{\prime \prime }\right)}\right)\right)}^{2} \end{array} \end{array}$$

where $i^{\prime }=2i^{\prime \prime }-1$ and $j^{\prime }=2i^{\prime \prime }$. One can verify that

(16) $$\begin{array}{c} Z\left(E_{\frac{N}{2^{n-m-1}}}^{\left(i^{\prime }\right)}\right)-Z\left(E_{\frac{N}{2^{n-m-1}}}^{\left(j^{\prime }\right)}\right)=2Z\left(E_{\frac{N}{2^{n-m}}}^{\left(i^{\prime \prime }\right)}\right)-{\left(Z\left(E_{\frac{N}{2^{n-m}}}^{\left(i^{\prime \prime }\right)}\right)\right)}^{2}-{\left(Z\left(E_{\frac{N}{2^{n-m-1}}}^{\left(i^{\prime \prime }\right)}\right)\right)}^{2} \end{array}$$

Since $Z\left(E_{\frac{N}{2^{n-m}}}^{\left(i^{\prime \prime }\right)}\right)$ takes values in $\left[0,1\right]$, we must have $Z\left(E_{\frac{N}{2^{n-m-1}}}^{\left(i^{\prime }\right)}\right)-Z\left(E_{\frac{N}{2^{n-m-1}}}^{\left(j^{\prime }\right)}\right)\geq 0$, with equality holds for $Z\left(E_{\frac{N}{2^{n-m}}}^{\left(i^{\prime \prime }\right)}\right)=0$ or 1.

Suppose the *i*th and the *j*th nodes in the $\left(m+2\right)$th CNOT gates block connect to the $i^{\prime }$th node ⊕ and the $j^{\prime }$th node ⊙ in the $\left(m+1\right)$th CNOT gates block, respectively, according to Equation (14), the index *i* and *j* satisfy $\left(i=2i^{\prime },\;j=2j^{\prime }\right)$ or $\left(i=2i^{\prime }-1,\;j=2j^{\prime }-1\right)$.

 **Case 1:**  if $i=2i^{\prime }$, $j=2j^{\prime }$, we have
  (17) $$\begin{array}{c} Z\left(E_{\frac{N}{2^{n-m-2}}}^{\left(i\right)}\right)=Z{\left(E_{\frac{N}{2^{n-m-1}}}^{\left(i^{\prime }\right)}\right)}^{2}\\ Z\left(E_{\frac{N}{2^{n-m-2}}}^{\left(j\right)}\right)=Z{\left(E_{\frac{N}{2^{n-m-1}}}^{\left(j^{\prime }\right)}\right)}^{2} \end{array}$$
 **Case 2:**  if $i=2i^{\prime }-1$, $j=2j^{\prime }-1$, we have

(18) $$\begin{array}{c} Z\left(E_{\frac{N}{2^{n-m-2}}}^{\left(i\right)}\right)=2Z\left(E_{\frac{N}{2^{n-m-1}}}^{\left(i^{\prime }\right)}\right)-Z{\left(E_{\frac{N}{2^{n-m-1}}}^{\left(i^{\prime }\right)}\right)}^{2}\\ Z\left(E_{\frac{N}{2^{n-m-2}}}^{\left(j\right)}\right)=2Z\left(E_{\frac{N}{2^{n-m-1}}}^{\left(j^{\prime }\right)}\right)-Z{\left(E_{\frac{N}{2^{n-m-1}}}^{\left(j^{\prime }\right)}\right)}^{2} \end{array}$$

Since $Z\left(E_{N/2^{n-m-1}}^{\left(i^{\prime }\right)}\right)\geq Z\left(E_{\frac{N}{2^{n-m-1}}}^{\left(j^{\prime }\right)}\right)$, no matter which case, we have,

(19) $$Z\left(E_{\frac{N}{2^{n-m-2}}}^{\left(i\right)}\right)>Z\left(E_{\frac{N}{2^{n-m-2}}}^{\left(j\right)}\right)$$

Using this recursion of the Bhattacharyya parameter, one can obtain

(20) $$Z\left(E_{N}^{\left(a\right)}\right)>Z\left(E_{N}^{\left(b\right)}\right)$$

which completes the proof.    □

It should be noted that when $n_{⊕}^{a}>n_{⊕}^{b}$, it is uncertain which of either $Z\left(E_{N}^{\left(a\right)}\right)$ or $Z\left(E_{N}^{\left(b\right)}\right)$ is greater.

Using Theorem 1 and Lemma 3, we can obtain Corollary 1, namely, the weight of stabilizer generators and logical X operators of QPSCs will polarize.

 **Corollary 1** **(Polarization of the weight of stabilizer generators and logical *X* operators of QPSCs).**  *For the encoding circuit $E_{N}$ as shown in Figure 2c, the corresponding stabilizer generators of quantum coordinate channels are $S_{1},\cdots ,S_{N}$, which can be divided into $n+1\;(n=\log_{2}N)$ parts according to their weight, namely,*

(21) $$S=\{S_{weight=2^{0}},S_{weight=2^{1}},\cdots ,S_{weight=2^{n}}\}$$

*where $S_{weight=2^{i}}\in S$ is the subset of S, which contains all stabilizer generators whose weight is $2^{i}$. Likewise, the corresponding logical X operators of quantum coordinate channels are $L=\{\bar{X_{1}},\cdots ,\bar{X_{N}}\}$ which can be divided into n+1 parts according to their weight, namely,*

(22) $$L=\{L_{weight=2^{0}},L_{weight=2^{1}},\cdots ,L_{weight=2^{n}}\}$$

*Since the channel quality will polarize, according to Lemma 3 and Theorem 1, the weight of stabilizer generators and logical X operators of QPSCs will polarize.*

 **Proof.**  For a path $P=\left\{{node}_{n},\cdots ,{node}_{1}\right\}$ in the encoding circuit $E_{N}$, where $n=\log_{2}N$ and ${node}_{i}\in ⨁,⨀$, 1≤i≤n, suppose the number of target node ⨁ of P is $n_{⊕}=x\;\left(0\leq x\leq n\right)$, by Lemma 3, its weight is $2^{x}$. Since each ${node}_{i}\;\left(1\leq i\leq n\right)$ only takes values in ⨁,⨀, and all paths in $E_{N}$ are unique, there are nx different paths whose number of target node ⨁ are *x*. Moreover, these nx different paths correspond to nx stabilizer generators whose weights are all $2^{x}$. Hence, the set $S=S_{1},\cdots ,S_{N}$ can be divided into n+1 parts according to the weight, namely, $S=\{S_{weight=2^{0}},S_{weight=2^{1}},\cdots ,S_{weight=2^{n}}\}$.

By Theorem 1, the Bhattacharyya parameter of the ni quantum coordinate channels whose corresponding ni stabilizer generators are all $2^{i}$ is lager than that of the nj quantum coordinate channels whose corresponding nj stabilizer generators are all $2^{j}$, for arbitrary 0≤j<i≤n. As the Bhattacharyya parameter will polarize, the weight of the stabilizer will polarize.

For the set of logical *X* operators $L=\{\bar{X_{1}},\cdots ,\bar{X_{N}}\}$, we have the same conclusion and the similar proof is omitted.    □

For a stabilizer code *C*, suppose its stabilizer group is $𝒮=\langle S_{1},\cdots ,S_{N-k}\rangle$, there is a useful way to represent 𝒮 by using **parity-check matrix** *H*. In bit-flip channels, all stabilizer generators of 𝒮 only contains Pauli *Z* operator, Hence *H* is a (N−k)×N matrix whose rows correspond to generators $S_{1}$ through $S_{N-k}$. For generator $S_{i}$, if it contains a *Z* on the *j*th qubit, the element $g_{i,j}$ of *H* located at the *i*th row and the *j*th column is 1, while it contains an *I* on the *j*th qubit, the element $g_{i,j}$ of *H* located at the *i*th row and the *j*th column is 0. For the logical operators of *C*, we can use a similar way, namely, **logical operator matrix** to represent it.

For QPSCs with code length $N=2^{n},n\geq 1$, if the *i*th input qubit $Q_{i}$ is a frozen qubit, the *i*th element of 1×N row vector representation $r\left(Z_{i}\right)$ of Pauli *Z* operator $Z_{i}$ acting on $Q_{i}$ is 1, while the rest of it is 0. The stabilizer generator $S_{i}$, which is transformed from $Z_{i}$ under conjugation by the encoding process $U_{E_{N}}$, is $S_{i}=U_{E_{N}}Z_{i}U_{E_{N}}^{†}$, and its corresponding row vector representation is

(23) $$r\left(S_{i}\right)=\;r\left(Z_{i}\right)G_{N}$$

where $G_{N}=F^{⊗n}$, $F=\left(\begin{array}{cc} 1 & 1\\ 0 & 1 \end{array}\right)$. Likewise, if $Q_{i}$ is a logical qubit, the Pauli *X* operator acting on $Q_{i}$ is $X_{i}$ and corresponding row vector representation is $r(X_{i})$. Then the logical *X* operator, which is transformed from $X_{i}$ under conjugation by the encoding process $U_{E_{N}}$, is $\bar{X_{i}}=U_{E_{N}}X_{i}U_{E_{N}}^{†}$, and its corresponding row vector representation is

(24) $$r\left(\bar{X_{i}}\right)=\;r\left(X_{i}\right)G_{N}^{T}$$

## 4. Construction Algorithms of Quantum Polar Stabilizer Codes

In this section, we propose two construction algorithms of QPSCs—CA algorithm and BS algorithm. The first algorithm is based on the ranking of channel quality of quantum coordinate channels which is similar to the code construction of classical polar code mentioned in Section 2.3. The second algorithm is based on the ranking of quantum coordinate channels according to the weight of their corresponding stabilizer generators. It is obvious that CA is the same as the construction in Ref. [33]. We believe BS is more reasonable than the existing constructions, because they are designed in a way from the point of view of stabilizer codes by considering the weight of logical operators and stabilizers and the code distance, which influence the correcting capability of stabilizer codes.

### 4.1. Coherent-Information-Achieving Construction

As mentioned in Section 2.4, the Bhattacharyya parameter can also be used to measure quantum channel quality, and we can approximately estimate the Bhattacharyya parameter of each $E_{N}^{\left(i\right)}$ by Equation (14). In CA algorithm, we use the Bhattacharyya parameter to rank channel quality. The detailed process of CA is as follows,

 **Step 1:**  For bit-flip channel E with error probability *p*, compute its symmetric coherent information (i.e., MSLCI) $I\left(\rho =\frac{1}{2}|0\rangle \langle 0|+\frac{1}{2}|1\rangle \langle 1|,E\right)=1-H\left(p\right)$, where H(·) is the Shannon entropy.
 **Step 2:**  Determine code length *N* and compute the number of logical qubits $k=\left\lfloor N(1-H(p))\right\rfloor$.
 **Step 3:**  Use Equation (14) to approximately estimate the Bhattacharyya parameter $Z(E_{N}^{\left(i\right)})$ of each $E_{N}^{\left(i\right)}$, then rank the coordinate channels in descending order according to their Bhattacharyya parameter and choose the former *k* channels to transmit data qubits while the last N−k channels transmit frozen qubits, which are set to state ${|0\rangle }^{⊗N-k}$.
 **Step 4:**  Use Equation (23) and Equation (24) to compute the row vector representation of stabilizer generators and logical operators.

The pseudocode is shown in Algorithm 1.
**Algorithm 1** Coherent-information-achieving construction **Require:** primal error rate *p*, code length parameter *n* **Ensure:** parity-check matrix *H*, logical operator matrix $H_{L}$ CodeLength $N=2^{n}$; $k=\left\lfloor N\times (1-H(p))\right\rfloor$; Compute the Bhattacharyya parameter of all quantum coordinate channels: $Z_{BP}$; $ChannelOrder=Sort(Z_{BP},\;‘descend\text{’})$; FrozenQubitsIndex=ChannelOrder(1:N−k); LogicalQubitsIndex=ChannelOrder(N−k+1:N); Initialize H=zeros(N−k,N), $H_{L}=zeros\left(k,N\right)$; **for** i=1 to N−k **do**    $H\left[i,FrozenQubitsIndex[i]\right]=1$ **end for** **for** i=1 to *k* **do**    $H_{L}\left[i,LogicalQubitsIndex[i]\right]=1$;    $H=HG_{N}$;    $H_{L}=H_{L}G_{N}^{T}$; **end for**

Under this construction algorithm, our simulation results in Section 5 show that the LERs increase with code lengths. It is obvious that the CA construction is equivalent to the construction in Ref. [33]. According to Theorem 1, one can understand why we choose the information position by channel quality—this will guarantee us to obtain a code with a distance as large as possible.

### 4.2. Block Selection Construction

According to Theorem 1, if the channel quality of the *i*th quantum coordinate channel is worse than that of the *j*th, the weight of the corresponding stabilizer generator of the *i*th must be no less than that of the *j*th. Using this, we can rank quantum coordinate channels according to the weight of their corresponding stabilizer generators in a block-by-block way. The second algorithm called block selection is based on this ranking.

For arbitrary stabilizer code, its code distance, which is defined as the minimum weight of its logical operators, determines the number of error qubits it can correct. Increasing its code distance will help to improve its error-correcting capability. Given a certain code length, more logical qubits (namely, less stabilizer generators) may lead to a smaller code distance. Hence, the goal of the block selection algorithm is to increase the number of stabilizer generators to increase the code distance of QPSCs.

For bit-flip channel E with error probability *p*, under the CA algorithm, the number of logical qubits of a QPSC is $k=\left\lfloor N(1-H(p))\right\rfloor$. We rank the quantum coordinate channels in descending order according to the weight of their corresponding stabilizer generators. Since there might be more than one coordinate channel with the same stabilizer generator weight, this ranking method will rank the coordinate channels block by block, and those coordinate channels in the same block have the same stabilizer generator weight. Then we assume that the weight of stabilizer generators of the $\left(N-k\right)$th quantum coordinate channel is $2^{x}\;\left(0\leq x\leq n=\log_{2}N\right)$. According to Lemma 3, the minimum weight of logical operators is $2^{n-x}$. Notice that there are nx quantum coordinate channels that the weight of their corresponding stabilizer generators is equal to $2^{x}$. If these nx channels are all chosen to transmit frozen qubits, the total number of stabilizer generators will be ∑m=xnnm, and the minimum weight of logical operators will increase to $2^{n-x+1}$. We can see that this can help to improve the error-correcting capability of QPSCs, since it increases the number of stabilizer generators and the code distance. Notice that we chose quantum coordinate channels in a block-wise way. If a coordinate channel is chosen to transmit frozen/data qubits, the whole block it belongs to will be chosen to transmit frozen/data. Hence, this algorithm is called block selection. The detailed process is as follows:

 **Step 1:**  For bit-flip channel E with error probability *p*, compute its symmetric coherent information $I\left(\rho =\frac{1}{2}|0\rangle \langle 0|+\frac{1}{2}|1\rangle \langle 1|,E\right)=1-H\left(p\right)$, where H(·) is the Shannon entropy.
 **Step 2:**  Determine code length *N* and compute $k^{\prime }=\left\lfloor N(1-H(p))\right\rfloor$. Different from the case in CA algorithm, we emphasize that $k^{\prime }$ here is not the number of logical qubits, but just an intermediate number which is used to determine the number of logical qubit *k*.
 **Step 3:**  Through Equation (23), compute the stabilizer generator of each quantum coordinate channel, and rank these channels in descending order according to the weight of their corresponding stabilizer generators.
 **Step 4:**  Find the $\left(N-k^{\prime }\right)$th quantum coordinate channel and computing the weight of its corresponding stabilizer generator. Suppose the weight is $2^{x}\;\left(0\leq x\leq n=\log_{2}N\right)$, then choose all quantum coordinate channels that the weight of their corresponding stabilizer generators is greater than or equal to $2^{x}$ to transmit frozen qubits. We can see that the number of frozen qubits is ∑a=xnna.
 **Step 5:**  Compute the number of logical qubits k=N−∑a=xnna, and the former N−k quantum coordinate channels are used to transmit frozen qubits which are set to state ${|0\rangle }^{⊗N-k}$ and the last *k* channels to transmit logical qubits.
 **Step 6:**  Use Equation (23) and Equation (24) to compute the row vector representation of each logical operator and stabilizer generator.

The pseudocode is shown in Algorithm 2.

One can see that given the same error probability *p*, the coding rate of CA is greater than or equal to that of BS, and the number of stabilizer generators and hence the code distance of CA is no more than that of BS. Thus, intuitively, the error-correcting capability of the BS algorithm is greater than or equal to that of the CA algorithm. However, the simulation results in Section 5 do not show this feature so obviously. This is because, in the range of error probability we consider, the average number of error qubits in a single simulation test is much lower than the code distance for both BS and CA. Hence, the error-correcting capabilities of the BS and CA algorithms are similar to each other.
**Algorithm 2** Block selection construction **Require:** primal error rate *p*, code length parameter *n* **Ensure:** parity-check matrix *H*, logical operator matrix $H_{L}$ CodeLength $N=2^{n}$;  $k^{\prime }=\left\lfloor N\times (1-H(p))\right\rfloor$;  Initialize S=zeros(N,N);  **for** i=1 to *N* **do**     $S\left[i,i\right]=1$  **end for** $S=SG_{N}$;  Compute the weight of all rows of *S*Weight;  ChannelOrder=Sort(Weight,‘descend’);  $w=ChannelOrder[k^{\prime }]$;  $x=\log_{2}w$;  k=∑a=xnna;  FrozenQubitsIndex=ChannelOrder(1:N−k);  LogicalQubitsIndex=ChannelOrder(N−k+1:N);  Initialize H=zeros(N−k,N), $H_{L}=zeros\left(k,N\right)$;  **for** i=1 to N−k **do**     $H\left[i,FrozenQubitsIndex[i]\right]=1$;  **end for**  **for** i=1 to *k* **do**     $H_{L}\left[i,LogicalQubitsIndex[i]\right]=1$;  **end for**$H=HG_{N}$;  $H_{L}=H_{L}G_{N}^{T}$;

## 5. Simulation Results and Analysis

There is an important concept of QECCs—noise threshold. For a certain class of QECCs, when the error probability is lower than its noise threshold, we can decrease the LER of a single logical qubit $LER_{slq}$ by increasing the code length. When the error probability is higher than the noise threshold, the $LER_{slq}$ will increase with the code length growth, which means this QECC fails. If a class of codes works for quantum computing, it should have such a threshold.

To test the error-correcting capability of QPSCs and find out whether they have such threshold, we perform simulations. In the range of *p* from $1\times 10^{-5}$ to $1\times 10^{-2}$, under both unreliable and reliable frozen qubits assumptions, no evidence shows that the LER of single logical qubit $LER_{slq}$ of both CA and BS can be decreased by increasing the code length and we cannot find the noise threshold. Notice that reliable frozen qubits correspond to reliable entanglement qubits in ref. [33].

### 5.1. Simulation Results

In the simulations, we assume that the error syndrome measurement is perfect and perform 100,000 simulations for each data point. Besides, two decoders are used. The first decoders is named table-look-up decoders which is realized by creating a syndrome-to-error lookup table that exhaustively list the best recovery operation for each error syndrome. The time cost and memory space of creating such complete syndrome-to-error lookup table rapidly become intractable as code length growing. To reduce the time cost and memory space, we only create an approximate incomplete table that lists the error whose the number of error qubits is within $t_{error}$, where $t_{error}$ is a parameter to control the approximation and larger than the average number of error qubits $Np_{0}$ in a single simulation test, where *N* is the code length, *p* is the qubit error rate. This is because in each simulation the probability of the events that the number of error qubits does not equal to $Np_{0}$ tends to zero as *N* grows. Hence, setting $t_{error}$ to a sufficient large value can guarantee the lookup table sufficiently complete for practical decoding task. We also use another decoder called bit-flip decoder whose time cost and memory space is linear with the code length. The simulation results show that in the low error rate regime from $1\times 10^{-5}$ to $1\times 10^{-2}$, the error-correcting performance of these two decoders is close. However, we observed that the bit-flip decoder is sensitive to the primal error rate *p*, and it may fail in the high error rate regime with code length increasing. The details of the bit-flip decoder are shown in Appendix A.

**Figure 4:**
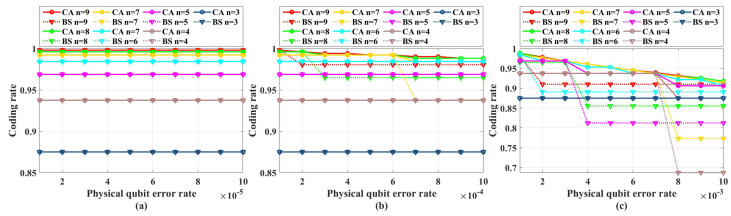
The coding rate of CA and BS algorithms with different physical qubit error rates and code lengths $N=2^{n}$. (**a**) Physical error rate ranges from $1\times 10^{-5}$ to $1\times 10^{-4}$. (**b**) Physical error rate ranges from $1\times 10^{-4}$ to $1\times 10^{-3}$. (**c**) Physical error rate ranges from $1\times 10^{-3}$ to $1\times 10^{-2}$.

Figure 4 shows the coding rate of the CA and BS algorithms with different physical qubit error rates and code lengths. We can see that in the range of *p* from $1\times 10^{-5}$ to $1\times 10^{-4}$, under different code lengths considered in the simulations, the coding rate of CA is the same as that of the BS algorithm. Hence, in the range of *p* from $1\times 10^{-5}$ to $1\times 10^{-4}$, we only need to perform simulations under CA algorithms.

**Figure 5:**
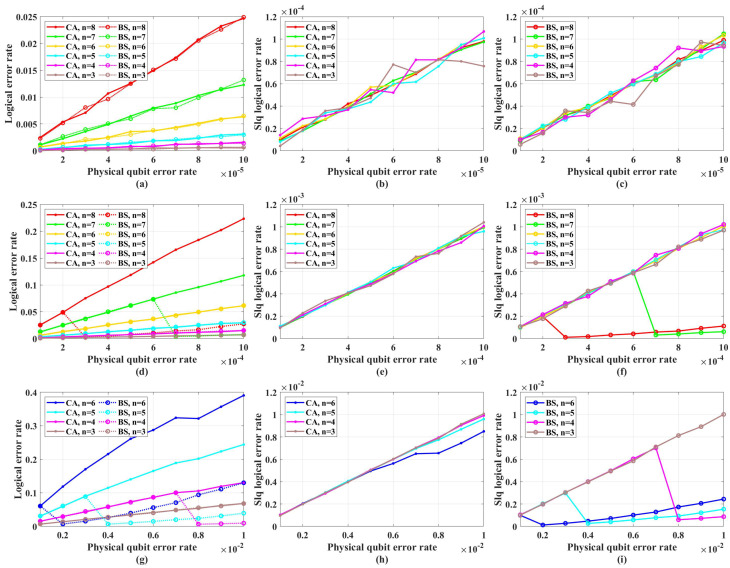
The LER with unreliable frozen qubits and table-look-up decoder. “Logical error rate” represents $LER_{block}$ and “Slq Logical error rate” represents $LER_{slq}$. (**a**) The $LER_{block}$ of QPSCs constructed by CA and BS algorithms with physical error rate ranging from $1\times 10^{-5}$ to $1\times 10^{-4}$. (**b**) The $LER_{slq}$ of QPSCs constructed by CA algorithms with physical error rate ranging from $1\times 10^{-5}$ to $1\times 10^{-4}$. (**c**) The $LER_{slq}$ of QPSCs constructed by BS algorithms with physical error rate ranging from $1\times 10^{-5}$ to $1\times 10^{-4}$. (**d**) The $LER_{block}$ of QPSCs constructed by CA and BS algorithms with physical error rate ranging from $1\times 10^{-4}$ to $1\times 10^{-3}$. (**e**) The $LER_{slq}$ of QPSCs constructed by CA algorithms with physical error rate ranging from $1\times 10^{-4}$ to $1\times 10^{-3}$. (**f**) The $LER_{slq}$ of QPSCs constructed by BS algorithms with physical error rate ranging from $1\times 10^{-4}$ to $1\times 10^{-3}$. (**g**) The $LER_{block}$ of QPSCs constructed by CA and BS algorithms with physical error rate ranging from $1\times 10^{-3}$ to $1\times 10^{-2}$. (**h**) The $LER_{slq}$ of QPSCs constructed by CA algorithms with physical error rate ranging from $1\times 10^{-3}$ to $1\times 10^{-2}$. (**i**) The $LER_{slq}$ of QPSCs constructed by BS algorithms with physical error rate ranging from $1\times 10^{-3}$ to $1\times 10^{-2}$.

**Figure 6:**
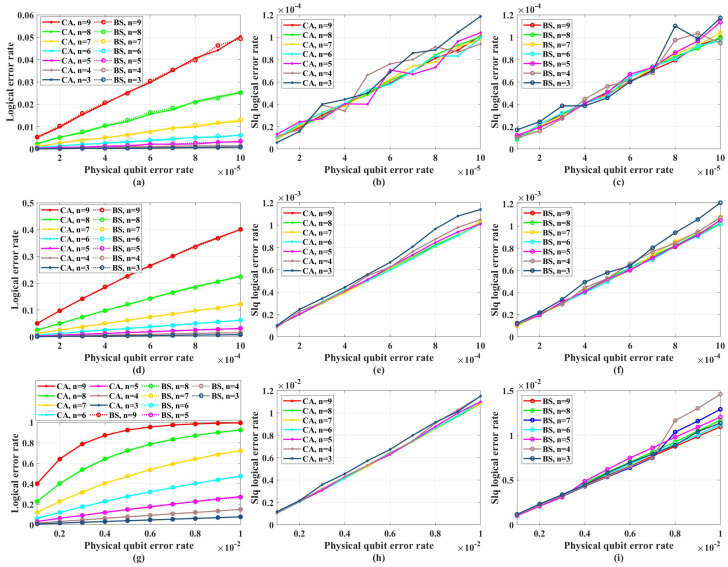
The LER with unreliable frozen qubits and bit-flip decoder. “Logical error rate” represents $LER_{block}$ and “Slq Logical error rate” represents $LER_{slq}$. (**a**) The $LER_{block}$ of QPSCs constructed by CA and BS algorithms with physical error rate ranging from $1\times 10^{-5}$ to $1\times 10^{-4}$. (**b**) The $LER_{slq}$ of QPSCs constructed by CA algorithms with physical error rate ranging from $1\times 10^{-5}$ to $1\times 10^{-4}$. (**c**) The $LER_{slq}$ of QPSCs constructed by BS algorithms with physical error rate ranging from $1\times 10^{-5}$ to $1\times 10^{-4}$. (**d**) The $LER_{block}$ of QPSCs constructed by CA and BS algorithms with physical error rate ranging from $1\times 10^{-4}$ to $1\times 10^{-3}$. (**e**) The $LER_{slq}$ of QPSCs constructed by CA algorithms with physical error rate ranging from $1\times 10^{-4}$ to $1\times 10^{-3}$. (**f**) The $LER_{slq}$ of QPSCs constructed by BS algorithms with physical error rate ranging from $1\times 10^{-4}$ to $1\times 10^{-3}$. (**g**) The $LER_{block}$ of QPSCs constructed by CA and BS algorithms with physical error rate ranging from $1\times 10^{-3}$ to $1\times 10^{-2}$. (**h**) The $LER_{slq}$ of QPSCs constructed by CA algorithms with physical error rate ranging from $1\times 10^{-3}$ to $1\times 10^{-2}$. (**i**) The $LER_{slq}$ of QPSCs constructed by BS algorithms with physical error rate ranging from $1\times 10^{-3}$ to $1\times 10^{-2}$.

**Figure 7:**
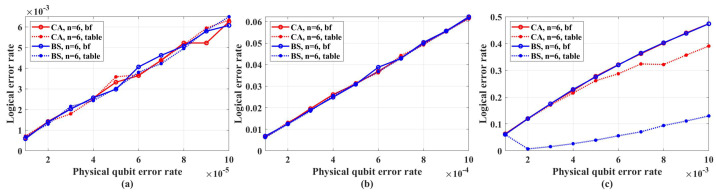
The performance of table-look-up decoder and bit-flip decoder in the range of *p* from $1\times 10^{-5}$ to $1\times 10^{-2}$. The code length is set to $N=2^{6}$. “Logical error rate” represents $LER_{block}$. (**a**) Physical error rate ranges from $1\times 10^{-5}$ to $1\times 10^{-4}$. (**b**) Physical error rate ranges from $1\times 10^{-4}$ to $1\times 10^{-3}$. (**c**) Physical error rate ranges from $1\times 10^{-3}$ to $1\times 10^{-2}$.

**Figure 8:**
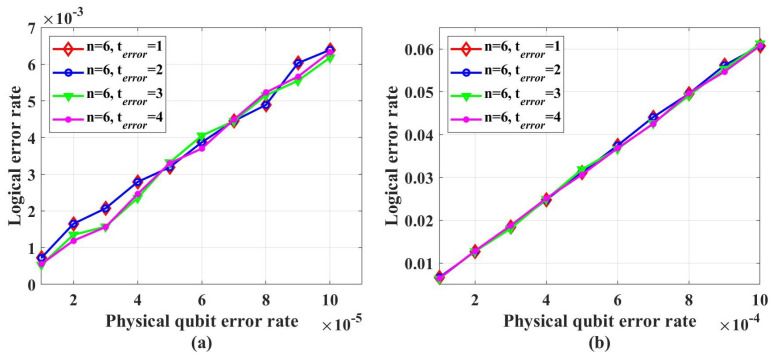
The performance of table-look-up decoder under CA algorithm with different $t_{error}$. “Logical error rate” represents $LER_{block}$. (**a**) Physical error rate ranges from $1\times 10^{-5}$ to $1\times 10^{-4}$. (**b**) Physical error rate ranges from $1\times 10^{-4}$ to $1\times 10^{-3}$.

**Figure 9:**
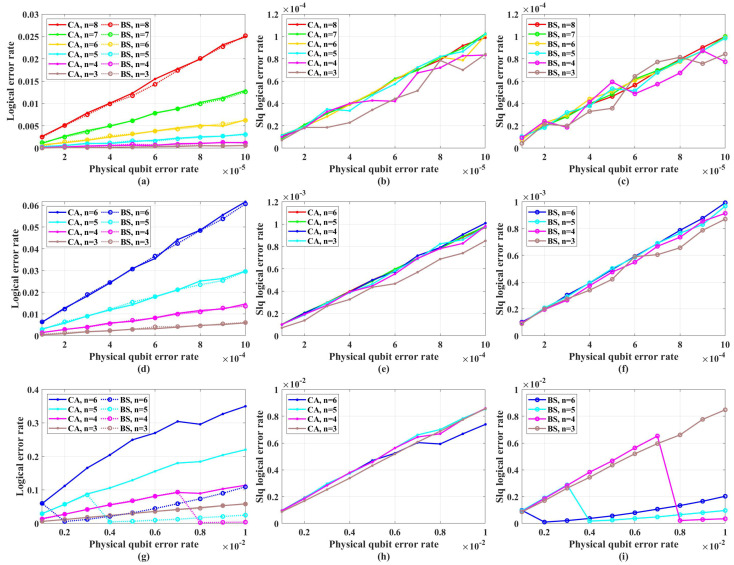
The LER with reliable frozen qubits and table-look-up decoder. “Logical error rate” represents $LER_{block}$ and “Slq Logical error rate” represents $LER_{slq}$. (**a**) The $LER_{block}$ of QPSCs constructed by CA and BS algorithms with physical error rate ranging from $1\times 10^{-5}$ to $1\times 10^{-4}$. (**b**) The $LER_{slq}$ of QPSCs constructed by CA algorithms with physical error rate ranging from $1\times 10^{-5}$ to $1\times 10^{-4}$. (**c**) The $LER_{slq}$ of QPSCs constructed by BS algorithms with physical error rate ranging from $1\times 10^{-5}$ to $1\times 10^{-4}$. (**d**) The $LER_{block}$ of QPSCs constructed by CA and BS algorithms with physical error rate ranging from $1\times 10^{-4}$ to $1\times 10^{-3}$. (**e**) The $LER_{slq}$ of QPSCs constructed by CA algorithms with physical error rate ranging from $1\times 10^{-4}$ to $1\times 10^{-3}$. (**f**) The $LER_{slq}$ of QPSCs constructed by BS algorithms with physical error rate ranging from $1\times 10^{-4}$ to $1\times 10^{-3}$. (**g**) The $LER_{block}$ of QPSCs constructed by CA and BS algorithms with physical error rate ranging from $1\times 10^{-3}$ to $1\times 10^{-2}$. (**h**) The $LER_{slq}$ of QPSCs constructed by CA algorithms with physical error rate ranging from $1\times 10^{-3}$ to $1\times 10^{-2}$. (**i**) The $LER_{slq}$ of QPSCs constructed by BS algorithms with physical error rate ranging from $1\times 10^{-3}$ to $1\times 10^{-2}$.

**Figure 10:**
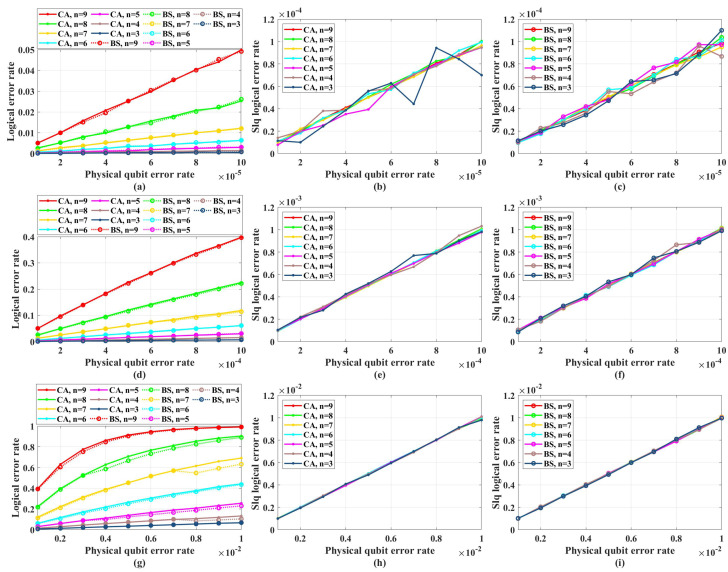
The LER with reliable frozen qubits and bit-flip decoder. “Logical error rate” represents $LER_{block}$ and “Slq Logical error rate” represents $LER_{slq}$. (**a**) The $LER_{block}$ of QPSCs constructed by CA and BS algorithms with physical error rate ranging from $1\times 10^{-5}$ to $1\times 10^{-4}$. (**b**) The $LER_{slq}$ of QPSCs constructed by CA algorithms with physical error rate ranging from $1\times 10^{-5}$ to $1\times 10^{-4}$. (**c**) The $LER_{slq}$ of QPSCs constructed by BS algorithms with physical error rate ranging from $1\times 10^{-5}$ to $1\times 10^{-4}$. (**d**) The $LER_{block}$ of QPSCs constructed by CA and BS algorithms with physical error rate ranging from $1\times 10^{-4}$ to $1\times 10^{-3}$. (**e**) The $LER_{slq}$ of QPSCs constructed by CA algorithms with physical error rate ranging from $1\times 10^{-4}$ to $1\times 10^{-3}$. (**f**) The $LER_{slq}$ of QPSCs constructed by BS algorithms with physical error rate ranging from $1\times 10^{-4}$ to $1\times 10^{-3}$. (**g**) The $LER_{block}$ of QPSCs constructed by CA and BS algorithms with physical error rate ranging from $1\times 10^{-3}$ to $1\times 10^{-2}$. (**h**) The $LER_{slq}$ of QPSCs constructed by CA algorithms with physical error rate ranging from $1\times 10^{-3}$ to $1\times 10^{-2}$. (**i**) The $LER_{slq}$ of QPSCs constructed by BS algorithms with physical error rate ranging from $1\times 10^{-3}$ to $1\times 10^{-2}$.

Figure 5 shows the simulation results with unreliable frozen qubits and table-look-up decoder. Figure 6 shows the simulation results with unreliable frozen qubits and the bit-flip decoder. It should be noticed that the LER of code blocks $LER_{block}$, which is the rate of code blocks with logical error, is directly obtained by counting the simulation results, while the LER of the single logical qubit $LER_{slq}$ is calculated from $LER_{block}$ by Equation (25), which is also used in [49]. These two decoders have similar performance in the range of *p* from $1\times 10^{-5}$ to $1\times 10^{-2}$, which is shown in Figure 7. In Figure 7, the parameter that is used to control the approximation accuracy is set to 4 in the range of *p* from $1\times 10^{-3}$ to $1\times 10^{-2}$ and 2 in the range of *p* from $1\times 10^{-5}$ to $1\times 10^{-3}$. In the physical qubit error rate range considered in our simulation, even for the largest code length ($N=2^{8}$ with physical qubit error rate less than $10^{-4}$ and $N=2^{6}$ with physical qubit error rate less than $10^{-2}$) we used, the average number of error qubits is less than 1, which means the probability of an error event that contains more than 1 error qubit generated by the simulation program is almost 0 (in our simulation, for 100,000 times test, only around one to three simulations will produce an error event contains more than one error qubit). The performance of the table-look-up decoder under the CA algorithm with different $t_{error}$ is shown in Figure 8. Figure 9 shows the simulation results with reliable frozen qubits and the table-look-up decoder. Figure 10 shows the simulation results with reliable frozen qubits and bit-flip decoder. As shown in Figure 5, Figure 6, Figure 9 and Figure 10, in the range of *p* from $1\times 10^{-5}$ to $1\times 10^{-2}$, there is no evidence showing that the LER of single logical qubit $LER_{slq}$ can be decreased by increasing the code length, and the noise threshold does not exist. (There are several points that seem abnormal in Figure 5d,f,g,i and Figure 10g,i. This is because of the abrupt change in the coding rate of BS algorithms.)

(25) $${\left(1-LER_{slq}\right)}^{k}=1-LER_{block}\;$$

Notice that the noise threshold we are talking about is the code-capacity threshold, which does not consider the noise in encoding and measurement. According to many other QLDPC codes such as surface code, the circuit-level noise threshold, which considers the noise in encoding and measurement, is often one–two orders lower than the code capacity threshold. This means even if the circuit-level noise threshold of QPSCs exists, it is lower than $10^{-6}$ in the best case. However, we believe this best case is a naïve case. Because, unlike surface code or other QLDPC codes, the stabilizer weight of QPSCs will increase with its code length, which means the encoding and measurement process will be influenced by the noise more greatly for larger code lengths. This will do harm to the circuit-level noise threshold. Hence, we believe that even if the circuit-level noise threshold of QPSCs exists, it is lower than $10^{-7}$ or even much lower.

**Figure 11:**
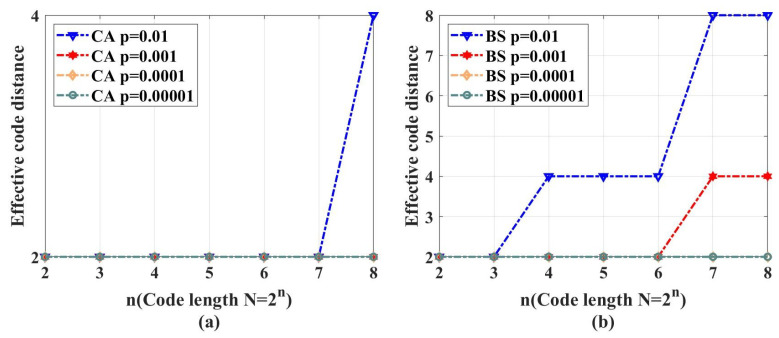
The effective code distance of CA and BS algorithms with different physical qubit error rates and code lengths $N=2^{n}$. (**a**) The effective code distance of QPSCs constructed by CA algorithms. (**b**) The effective code distance of QPSCs constructed by BS algorithms.

In fact, no one can exhaust all possible physical qubit error rates to prove whether the noise threshold exists. Hence, analyzing the code distance is relatively more powerful to judge whether a quantum stabilizer code can work or not. In our simulation, the weight of the logical *X* operators can be regarded as the effective code distance, because we only consider the Pauli *X* noise. Figure 11 shows the effective code distance of QPSCs constructed by CA and BS algorithms. Careful readers may notice that, given the same physical qubit error rate and code length, the effective code distance of CA may not be half of that of BS, which is not consistent with what we discuss in Section 4.2. This is because in CA algorithm the method used to estimate the Bhattacharyya parameter of the quantum coordinate channels is an approximate method, resulting that some chosen quantum coordinate channels are not consistent with the most reliable ones in theory, such that the corresponding effective code distance is lower than the theoretical value. This will not influence our judgment about the error-correcting capability, because we have clarified that the code distance of QPSCs constructed by CA algorithms will not surpass that obtained by BS algorithms.

As shown in Figure 11, we find that both the CA and BS construction algorithms cannot guarantee the code distance increase with the code length. This means that under the same physical qubit error rate, with the code length increasing, the average number of error qubits will increase, but the number of qubits this code can correct, which is determined by the code distance, does not increase with a sufficient rate and is almost invariant in low physical qubit error rate. That is to say, increasing the code length will only impair the code’s error-correcting capability and enlarge the logical error rate.

Notice that no matter for CA or BS construction algorithms, given a certain physical error rate and a certain code length, they both try to construct codes with a coding rate as high as possible according to the channel polarization theory. Though BS takes the code distance into consideration to some extent, it still regards the high coding rate as its primal target. Therefore, the effective code distance will be influenced by the physical error rate, instead of being determined by the code length alone. For example, in Figure 11, under the code distance $N=2^{7}$, when the physical qubit error rate *p* decreases from 0.01 to 0.001, the code distance decreases from 8 to 4 for the BS algorithm. The above characteristic of the effective code distance of the QPSCs constructed by CA and BS algorithms is the direct reason why the noise threshold does not exist.

### 5.2. Analysis

Why do QPSCs not work for quantum computing? We think this is due to the decoding channels and coordinate channels having no one-to-one relationships.

**Figure 12:**
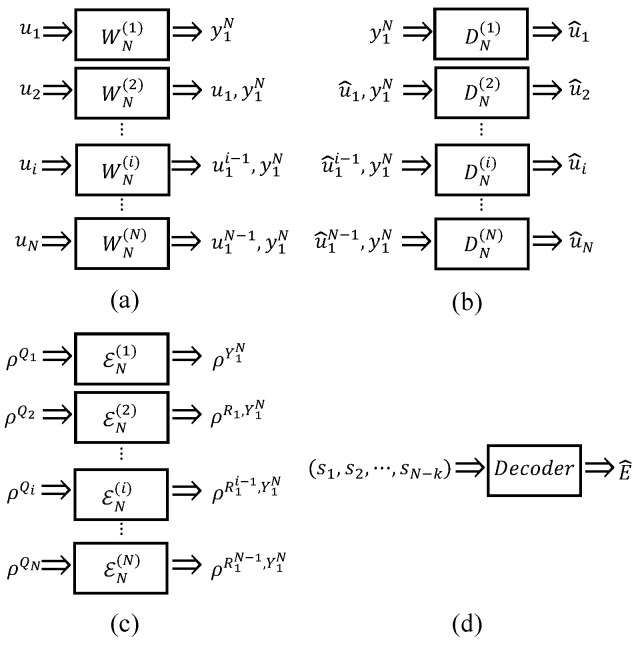
Coordinate channels and decoding channels: (**a**) Classical coordinate channels. The input of classical coordinate channel $W_{N}^{\left(i\right)}$ is $u_{i}$, and its output is $y_{1}^{N},u_{1}^{i-1}$. (**b**) Classical decoding channels. The input of decoding channel $D_{N}^{\left(i\right)}$ is ${\hat{u}}_{1}^{i-1},y_{1}^{N}$ and the output is estimated ${\hat{u}}_{i}$. (**c**) Quantum coordinate channels. The input of quantum coordinate channel $E_{N}^{\left(i\right)}$ is $\rho^{Q_{i}}$, and its output is $\rho^{Y_{1}^{N},R_{1}^{i-1}}$. (**d**) Quantum decoding channel. The input of decoding channel is an error syndrome and the output is the most likely error.

For classical polar codes, as shown in Figure 12a, the input of the classical coordinate channel $W_{N}^{\left(i\right)}$ is $u_{i}$, and its output is $y_{1}^{N},u_{1}^{i-1}$. During the decoding procedure, as shown in Figure 12b, the decoder estimates ${\hat{u}}_{i}$ after observing $y_{1}^{N}$ and the past estimated channel inputs ${\hat{u}}_{1}^{i-1}$. Hence, we can see that the classical polar code construction builds a one-to-one relationship between the classical coordinate channels and decoding channels. The better the quality of the coordinate channels are, the more reliable the decoding channels will be.

For QPSCs, as shown in Figure 12c, the input of the quantum coordinate channel $E_{N}^{\left(i\right)}$ is $\rho^{Q_{i}}$, and its output is $\rho^{Y_{1}^{N},R_{1}^{i-1}}$. However, for the quantum decoding channel as shown in Figure 12d, its input is an error syndrome and its output is the most likely error. Both CA and BS construction algorithms do not build a similar one-to-one relationship between the quantum coordinate channels and the decoding channel.

## 6. Possible Future Direction

**Figure 13:**
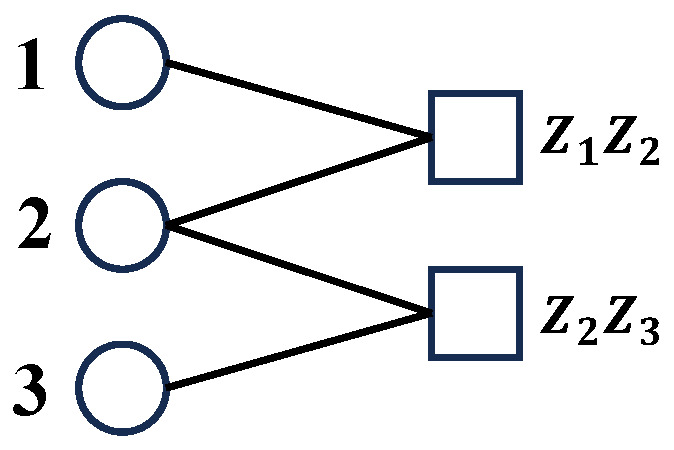
The Tanner graph of 3-bit-flip code.

Based on the analysis in Section 5.2, we argue that if researchers still try to borrow the idea of classical polar codes to design quantum stabilizer codes with high coding rate, the channels waiting to be analyzed and polarized should be the channels with the actual error on physical qubits as input and error syndrome as output, which corresponds to the channels in Figure 12d. What do these channels look like? The channels between the actual error on physical qubits and error syndrome can be depicted by the Tanner graph [50] as shown in Figure 13. However, it does not seem direct to polarize the Tanner graph by borrowing the idea of classical polar codes from the perspective of channel capacity, since the number of inputs (variable nodes) and outputs (check nodes) is different and each output may connect to more than one input, which leads to the difficulty in defining the primal channel used to realize polarization and analyzing the channel capacity.

**Figure 14:**
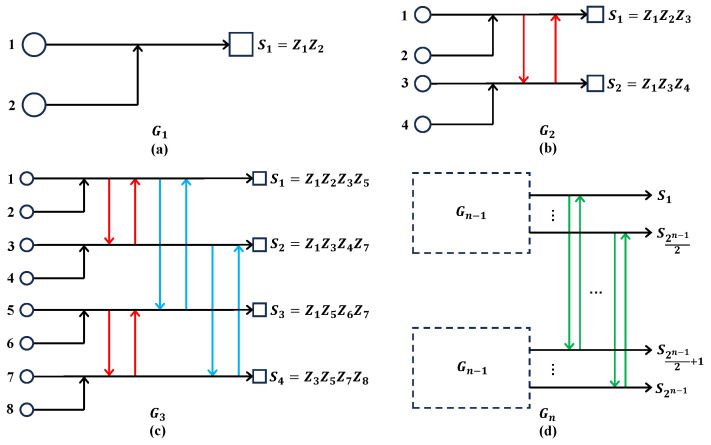
Quantum stabilizer codes by the recursive expansion of Tanner graph. The arrow means the corresponding qubit it starts from will join in the corresponding stabilizer it ends with. The corresponding stabilizers and logical operators are shown in Table 1: (**a**) The Tanner graph $G_{1}$ used for recursive expansion. (**b**) The expanded Tanner graph $G_{2}$ by recursive expansion of two $G_{1}$. (**c**) The expanded Tanner graph $G_{3}$ by recursive expansion of two $G_{2}$. (**d**) The expanded Tanner graph $G_{n}$ by recursive expansion of two $G_{n-1}$.

Arikan [11] uses the concept of channel capacity to design the recursive encoding of classical polar codes. It does no harm to put the concept of channel capacity of classical polar codes aside for a moment and focus on the recursive encoding itself. By recursive encoding, one can expand the encoding circuits and obtain classical polar codes with longer code lengths and stronger error-correcting capability from two shorter classical polar codes. Hence, can we recursively expand the Tanner graph of certain stabilizer codes by the idea of recursive encoding and obtain stabilizer codes with longer code lengths and stronger error-correcting capability? We have made some attempts in pure Pauli *X* noise channel and propose a class of stabilizer codes with a constant coding rate of 0.5 by recursively expanding the Tanner graph, which is shown in Figure 14. The scheme also applies to pure Pauli *Z* and Y noise channels.

As shown in Figure 14, the recursive expansion of the Tanner graph has the same form as the recursive encoding circuits of classical polar codes (Figure 8 in ref. [11]), and the coding rate of the corresponding code is the constant 0.5. For the minimum weight of logical X operators, if logN is an even number, it is logN, while if logN is an odd number, it is logN+1 (notice that in pure Pauli *X* noise, the error-correcting capability relies on the minimum weight of logical X operators, rather than the minimum weight of all logical operators, namely, the code distance).

It is easy to prove the coding rate and the minimum weight of logical X operators. The code length $N=2^{n}$, and notice that when *n* is an odd number, each stabilizer generator contains a unique Pauli *Z* operator with an even subscript, while when *n* is an even number, each stabilizer generator contains a unique Pauli *Z* operator with an odd subscript. Hence, each stabilizer generator can’t be generated by other stabilizer generators, which means that when the code length is $N=2^{n}$, the $2^{n-1}$ stabilizer generators in Figure 14d are independent. Therefore, the number of logical operators is $2^{n}-2^{n-1}=2^{n-1}$, which means the coding rate is 0.5. As for the minimum weight of logical X operators and stabilizer generators, one can easily prove this by mathematical induction from the code length N=4 to $N=2^{n}$.

**Figure 15:**
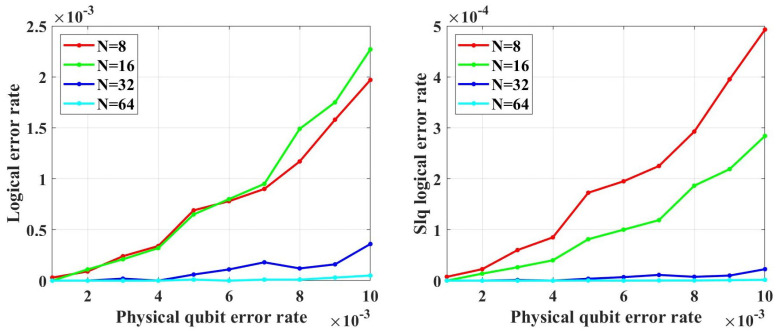
The LER with table-look-up decoder.

**Figure 16:**
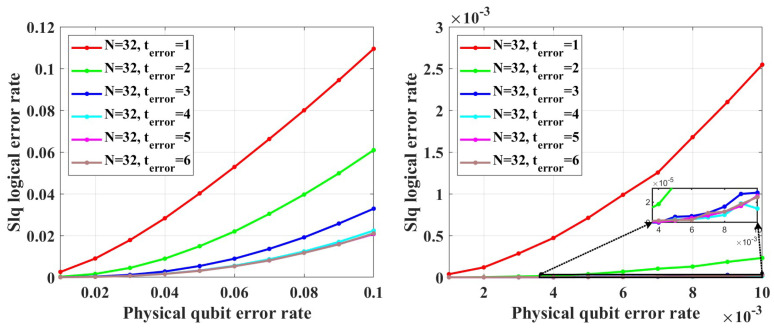
The decoding accuracy under different $t_{error}$.

To test the error-correcting capability of the codes, simulations with the table-look-up decoder are performed, whose results are shown in Figure 15. Figure 15 shows that the logical error rate will decrease with the increase in code length within the physical qubit error rate from 0.001 to 0.01. In the simulations, $t_{error}$ is set to 5. However, due to the complexity of the table-look-up decoder, we did not find the noise threshold of these codes. To find the noise threshold, simulations need to be performed at a larger physical qubit error rate. With the increase in physical qubit error rate, the average number of error qubits will increase. According to the results in Figure 16, for this class of codes, when $t_{error}$ is less than ten times the average number of error qubits, the decoding accuracy is sensitive to $t_{error}$. Hence, to perform simulations under a larger physical qubit error rate, $t_{error}$ needs to take a larger value, which leads to the required memory becoming intractable.

The above attempts are made for Pauli *X*. It is obvious that it also applies to Pauli *Y* errors. And simply replacing Pauli *Z* in the stabilizers by Pauli *X* or *Y* can provide us code that apply to Pauli *Z* and *Y* errors or Pauli *X* and *Z* errors. Whether it can be generalized to correct all three Pauli errors is still unknown. To make this generalization, maybe one should modify the primal Tanner graph and the recursive expansion approach. However, even without this generalization, we can still obtain codes that apply to depolarizing channels by concatenating two codes—one corrects the Pauli *X* and *Y* errors, and the other corrects Pauli *Z* and *Y* errors.

## 7. Conclusions

In this paper, we follow the intuition that one can design a quantum polar coding scheme for quantum computing based on the polarization phenomenon of quantum channels that arises from directly converting the classical polar coding circuits into quantum ones, and we propose two possible and more reasonable construction algorithms—the CA and BS algorithms—to construct QSPCs. The CA construction is equivalent to the construction in ref. [33]. In the range of *p* from $1\times 10^{-5}$ to $1\times 10^{-2}$, under both unreliable and reliable frozen qubits assumptions, no evidence shows that the LER of a single logical qubit $LER_{slq}$ of both CA and BS can be decreased by increasing the code length and we cannot find the noise threshold. Therefore, we come to the conclusion that the above intuition is too naive to guide us to design a quantum polar coding scheme for quantum computing. We also provide a possible future direction for designing quantum stabilizer codes with high coding rates by borrowing the idea of classical polar codes. Following this direction, we find a class of quantum stabilizer codes with a constant coding rate of 0.5, which can correct two kinds of Pauli errors.

## Figures and Tables

**Table 1:** The stabilizer generators and corresponding logical operators when code length N=4,8,16,32.

Code Length	Stabilizer Generators	Logical X Operators	Logical Z Operators
4	$S_{1}=Z_{1}Z_{2}Z_{3}$	${\bar{X}}_{1}=X_{1}X_{2}X_{4}$	${\bar{Z}}_{1}=Z_{1}$
	$S_{2}=Z_{1}Z_{3}Z_{4}$	${\bar{X}}_{2}=X_{2}X_{3}X_{4}$	${\bar{Z}}_{2}=Z_{3}$
8	$S_{1}=Z_{1}Z_{2}Z_{3}Z_{5}$	${\bar{X}}_{1}=X_{1}X_{2}X_{3}X_{5}$	${\bar{Z}}_{1}=Z_{2}$
	$S_{2}=Z_{1}Z_{3}Z_{4}Z_{7}$	${\bar{X}}_{2}=X_{1}X_{3}X_{4}X_{7}$	${\bar{Z}}_{2}=Z_{4}$
	$S_{3}=Z_{1}Z_{5}Z_{6}Z_{7}$	${\bar{X}}_{3}=X_{1}X_{5}X_{6}X_{7}$	${\bar{Z}}_{3}=Z_{6}$
	$S_{4}=Z_{3}Z_{5}Z_{7}Z_{8}$	${\bar{X}}_{4}=X_{3}X_{5}X_{7}X_{8}$	${\bar{Z}}_{4}=Z_{8}$
16	$S_{1}=Z_{1}Z_{2}Z_{3}Z_{5}Z_{9}$	${\bar{X}}_{1}=X_{1}X_{2}X_{4}X_{6}X_{10}$	${\bar{Z}}_{1}=Z_{1}$
	$S_{2}=Z_{1}Z_{3}Z_{4}Z_{7}Z_{11}$	${\bar{X}}_{2}=X_{2}X_{3}X_{4}X_{8}X_{12}$	${\bar{Z}}_{2}=Z_{3}$
	$S_{3}=Z_{1}Z_{5}Z_{6}Z_{7}Z_{13}$	${\bar{X}}_{3}=X_{2}X_{5}X_{6}X_{8}X_{14}$	${\bar{Z}}_{3}=Z_{5}$
	$S_{4}=Z_{3}Z_{5}Z_{7}Z_{8}Z_{15}$	${\bar{X}}_{4}=X_{4}X_{6}X_{7}X_{8}X_{16}$	${\bar{Z}}_{4}=Z_{7}$
	$S_{5}=Z_{1}Z_{9}Z_{10}Z_{11}Z_{13}$	${\bar{X}}_{5}=X_{2}X_{9}X_{10}X_{12}X_{14}$	${\bar{Z}}_{5}=Z_{9}$
	$S_{6}=Z_{3}Z_{9}Z_{11}Z_{12}Z_{15}$	${\bar{X}}_{6}=X_{4}X_{10}X_{11}X_{12}X_{16}$	${\bar{Z}}_{6}=Z_{11}$
	$S_{7}=Z_{5}Z_{9}Z_{13}Z_{14}Z_{15}$	${\bar{X}}_{7}=X_{6}X_{10}X_{13}X_{14}X_{16}$	${\bar{Z}}_{7}=Z_{13}$
	$S_{8}=Z_{7}Z_{11}Z_{13}Z_{15}Z_{16}$	${\bar{X}}_{8}=X_{8}X_{12}X_{14}X_{15}X_{16}$	${\bar{Z}}_{8}=Z_{15}$
32	$S_{1}=Z_{1}Z_{2}Z_{3}Z_{5}Z_{9}Z_{17}$	${\bar{X}}_{1}=X_{1}X_{2}X_{3}X_{5}X_{9}X_{17}$	${\bar{Z}}_{1}=Z_{2}$
	$S_{2}=Z_{1}Z_{3}Z_{4}Z_{7}Z_{11}Z_{19}$	${\bar{X}}_{2}=X_{1}X_{3}X_{4}X_{7}X_{11}X_{19}$	${\bar{Z}}_{2}=Z_{4}$
	$S_{3}=Z_{1}Z_{5}Z_{6}Z_{7}Z_{13}Z_{21}$	${\bar{X}}_{3}=X_{1}X_{5}X_{6}X_{7}X_{13}X_{21}$	${\bar{Z}}_{3}=Z_{6}$
	$S_{4}=Z_{3}Z_{5}Z_{7}Z_{8}Z_{15}Z_{23}$	${\bar{X}}_{4}=X_{3}X_{5}X_{7}X_{8}X_{15}X_{23}$	${\bar{Z}}_{4}=Z_{8}$
	$S_{5}=Z_{1}Z_{9}Z_{10}Z_{11}Z_{13}Z_{25}$	${\bar{X}}_{5}=X_{1}X_{9}X_{10}X_{11}X_{13}X_{25}$	${\bar{Z}}_{5}=Z_{10}$
	$S_{6}=Z_{3}Z_{9}Z_{11}Z_{12}Z_{15}Z_{27}$	${\bar{X}}_{6}=X_{3}X_{9}X_{11}X_{12}X_{15}X_{27}$	${\bar{Z}}_{6}=Z_{12}$
	$S_{7}=Z_{5}Z_{9}Z_{13}Z_{14}Z_{15}Z_{29}$	${\bar{X}}_{7}=X_{5}X_{9}X_{13}X_{14}X_{15}X_{29}$	${\bar{Z}}_{7}=Z_{14}$
	$S_{8}=Z_{7}Z_{11}Z_{13}Z_{15}Z_{16}Z_{31}$	${\bar{X}}_{8}=X_{7}X_{11}X_{13}X_{15}X_{16}X_{31}$	${\bar{Z}}_{8}=Z_{16}$
	$S_{9}=Z_{1}Z_{17}Z_{18}Z_{19}Z_{21}Z_{25}$	${\bar{X}}_{9}=X_{1}X_{17}X_{18}X_{19}X_{21}X_{25}$	${\bar{Z}}_{9}=Z_{18}$
	$S_{10}=Z_{3}Z_{17}Z_{19}Z_{20}Z_{23}Z_{27}$	${\bar{X}}_{10}=X_{3}X_{17}X_{19}X_{20}X_{23}X_{27}$	${\bar{Z}}_{10}=Z_{20}$
	$S_{11}=Z_{5}Z_{17}Z_{21}Z_{22}Z_{23}Z_{29}$	${\bar{X}}_{11}=X_{5}X_{17}X_{21}X_{22}X_{23}X_{29}$	${\bar{Z}}_{11}=Z_{22}$
	$S_{12}=Z_{7}Z_{19}Z_{21}Z_{23}Z_{24}Z_{31}$	${\bar{X}}_{12}=X_{7}X_{19}X_{21}X_{23}X_{24}X_{31}$	${\bar{Z}}_{12}=Z_{24}$
	$S_{13}=Z_{9}Z_{17}Z_{25}Z_{26}Z_{27}Z_{29}$	${\bar{X}}_{13}=X_{9}X_{17}X_{25}X_{26}X_{27}X_{29}$	${\bar{Z}}_{13}=Z_{26}$
	$S_{14}=Z_{11}Z_{19}Z_{25}Z_{27}Z_{28}Z_{31}$	${\bar{X}}_{14}=X_{11}X_{19}X_{25}X_{27}X_{28}X_{31}$	${\bar{Z}}_{14}=Z_{28}$
	$S_{15}=Z_{13}Z_{21}Z_{25}Z_{29}Z_{30}Z_{31}$	${\bar{X}}_{15}=X_{13}X_{21}X_{25}X_{29}X_{30}X_{31}$	${\bar{Z}}_{15}=Z_{30}$
	$S_{16}=Z_{15}Z_{23}Z_{27}Z_{29}Z_{31}Z_{32}$	${\bar{X}}_{16}=X_{15}X_{23}X_{27}X_{29}X_{31}X_{32}$	${\bar{Z}}_{16}=Z_{32}$

**Figure A1:**
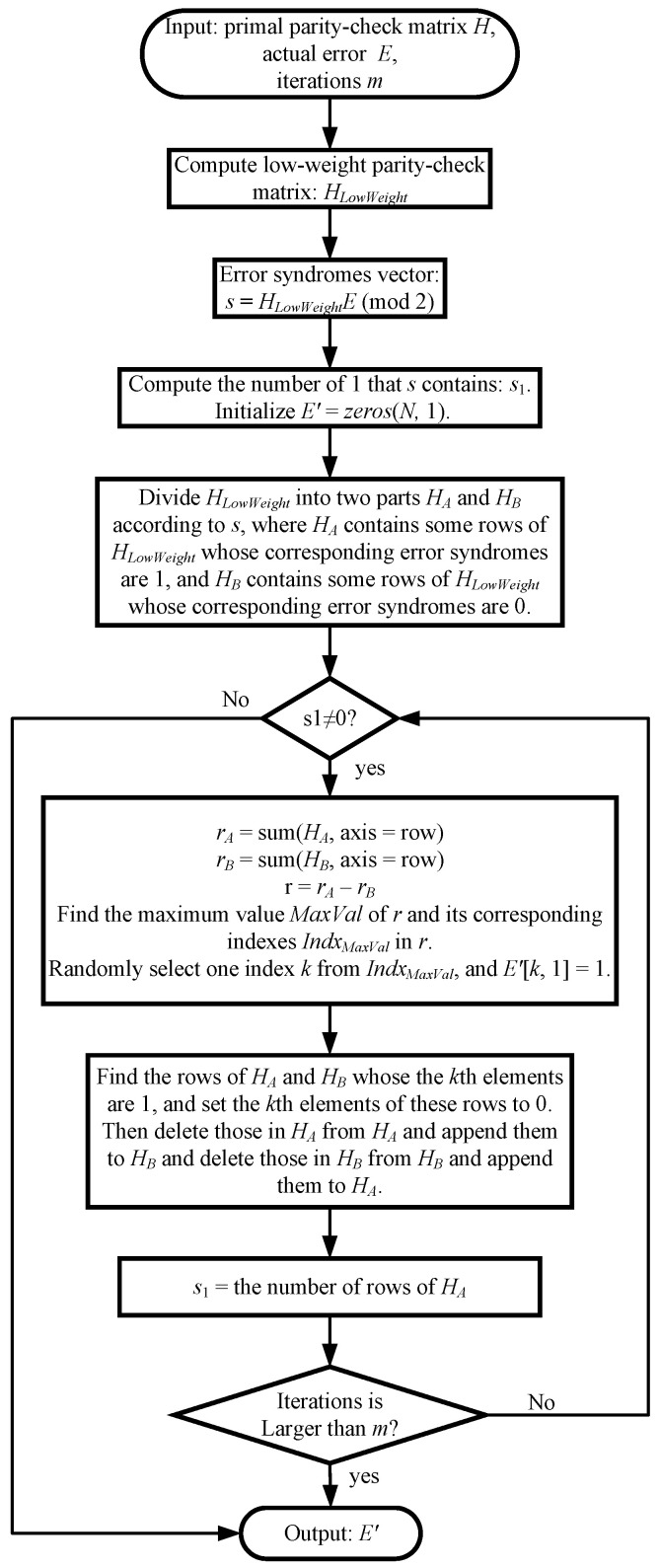
The flow chart corresponds to Algorithm 3.

## Data Availability

All the data generated or analyzed during this study are included in this article.

## Abbreviations

The following abbreviations are used in this manuscript:

BS	Block-selection
B-DMC	Binary-input discrete memoryless channel
BTPM	Basis transition probability matrix
CLDPC	Classical low-density parity check
CA	Coherent-information-achieving
CECC	Classical error correction codes
LER	Logical error rate
MSLCI	Maximum single-letter coherent information
QEC	Quantum error correction
QECC	Quantum error correction codes
QLDPC	Quantum low-density parity check
QSC	Quantum symmetric channel
QQSC	Quantum quasi symmetric channel
QPSC	Quantum polar stabilizer code
TPM	Transition probability matrix

## Appendix A. Bit-Flip Decoder

Here, we give the pseudocode of the bit-flip decoder, as shown in Algorithm 3. The corresponding flow chart is shown in Figure A1.

**Algorithm A1** Bit-flip decoder
**Require:** primal parity-check matrix *H*, actual error *E*, iterations *m* **Ensure:** estimated error $E^{\prime }$ Reduce the weight of each row of *H* using the elementary row transformation of matrix and obtain new parity-check matrix $H_{LowWeight}$; Error syndromes vector: $s=H_{LowWeight}E\;\left(mod\;2\right)$; Compute the number of 1 that *s* contains: $s_{1}$. Initialize $E^{\prime }=zeros\left(N,1\right)$; Divide $H_{LowWeight}$ into two parts $H_{A}$ and $H_{B}$ according to *s*, where $H_{A}$ contains some rows of $H_{LowWeight}$ whose corresponding error syndromes are 1, and $H_{B}$ contains some rows of $H_{LowWeight}$ whose corresponding error syndromes are 0; **while** $s_{1}\neq 0$ or m≠0 **do** $r_{A}=sum\left(H_{A},\;axis=row\right)$; $r_{B}=sum\left(H_{B},\;axis=row\right)$; $r=r_{A}-r_{B}$; Find the maximum value MaxVal of *r* and its corresponding indexes $Indx_{MaxVal}$ in *r*; Randomly select one index *k* from $Indx_{MaxVal}$; $E^{\prime }\left[k,1\right]=1$; **for** each row $H_{A}^{i}\in H_{A}$ **do** **if** $H_{A}^{i}\left[k\right]=1$ **then** $H_{A}^{i}\left[k\right]=0$; Delete $H_{A}^{i}$ from $H_{A}$ and append $H_{A}^{i}$ to $H_{B}$; **end if** **end for** **for** each row $H_{B}^{j}\in H_{B}$ **do** **if** $H_{B}^{j}\left[k\right]=1$ **then** $H_{B}^{j}\left[k\right]=0$; Delete $H_{B}^{j}$ from $H_{B}$ and append $H_{B}^{j}$ to $H_{A}$; **end if** **end for** $s_{1}=the\;number\;of\;rows\;of\;H_{A}$; m=m−1; Output $E^{\prime }$; **end whole**
